# Identification of human age-associated gene co-expressions in functional modules using liquid association

**DOI:** 10.18632/oncotarget.23148

**Published:** 2017-12-08

**Authors:** Jialiang Yang, Yufang Qin, Tiantian Zhang, Fayou Wang, Lihong Peng, Lijuan Zhu, Dawei Yuan, Pan Gao, Jujuan Zhuang, Zhongyang Zhang, Jun Wang, Yun Fang

**Affiliations:** ^1^ College of Information Engineering, Changsha Medical University, Changsha, Hunan, P. R. China; ^2^ Department of Mathematics, Shanghai Ocean University, Shanghai, China; ^3^ School of Mathematics and Information Science, Henan Polytechnic University, Henan, P. R. China; ^4^ Department of Mathematics, Hebei University of Science and Technology, Shijiazhuang, Hebei, China; ^5^ Geneis (Beijing) Co. Ltd., Beijing, P. R. China; ^6^ Department of Mathematics, Dalian Maritime University, Dalian, Liaoning, P. R. China; ^7^ Department of Genetics and Genomic Sciences, Icahn School of Medicine at Mount Sinai, New York, NY, USA; ^8^ Icahn Institute for Genomics and Multiscale Biology, Icahn School of Medicine at Mount Sinai, New York, NY, USA; ^9^ Department of Mathematics, Shanghai Normal University, Shanghai, P. R. China

**Keywords:** aging, anti-aging drug prediction, gene co-expression, liquid association, GTEx

## Abstract

Aging is a major risk factor for age-related diseases such as certain cancers. In this study, we developed Age Associated Gene Co-expression Identifier (AAGCI), a liquid association based method to infer age-associated gene co-expressions at thousands of biological processes and pathways across 9 human tissues. Several hundred to thousands of gene pairs were inferred to be age co-expressed across different tissues, the genes involved in which are significantly enriched in functions like immunity, ATP binding, DNA damage, and many cancer pathways. The age co-expressed genes are significantly overlapped with aging genes curated in the GenAge database across all 9 tissues, suggesting a tissue-wide correlation between age-associated genes and co-expressions. Interestingly, age-associated gene co-expressions are significantly different from gene co-expressions identified through correlation analysis, indicating that aging might only contribute to a small portion of gene co-expressions. Moreover, the key driver analysis identified biologically meaningful genes in important function modules. For example, *IGF1, ERBB2, TP53 and STAT5A* were inferred to be key genes driving age co-expressed genes in the network module associated with function “T cell proliferation”. Finally, we prioritized a few anti-aging drugs such as metformin based on an enrichment analysis between age co-expressed genes and drug signatures from a recent study. The predicted drugs were partially validated by literature mining and can be readily used to generate hypothesis for further experimental validations.

## INTRODUCTION

Aging is an inevitable, intrinsic and irreversible process, which not only means increase in age, but also leads to deterioration of physiological functions. During the aging process, individuals lose viability and simultaneously increase vulnerability. On the other hand, aging is also a major risk factor for a variety of human diseases. The incidences of a number of diseases increase with age including certain cancers, cardiovascular diseases, type II diabetes, Parkinson’s disease, Alzheimer’s disease, arthritis, and so on [1, 2]. As a result, aging study contributes to both human longevity and health.

As usually a first step towards aging study, identification of reliable aging biomarkers is critical to revealing the secrets behind aging as well as identifying drugs for longevity and age-related diseases (ARDs). Different types of biomarkers have been proposed to quantify human aging, varying from physical parameters like visual acuity, grey hair, and skin wrinkles [3] to molecular biomarkers like telomere length, gene expressions, and methylations [4, 5]. For example, the association between epigenetic variation (e.g., DNA methylation and histone modification) and age has been reported [6]. In addition, gene expression and methylation profiles of blood [5, 7, 8], gene expression profile of brain [9], and telomere length [10, 11] are good indicators for age in human and other primates. Recently, using GTEx pilot phase data, Yang et al. identified age-associated genes for 9 human tissues and showed an intimate association between aging and ARDs at gene expression level [4].

However, despite many important findings, previous studies are mostly focused on single biomarkers, e.g. single gene expressions and methylations. The binary relationships between biomarkers (e.g., gene co-expressions and gene regulations) perturbed by aging are more or less ignored. It is known that co-expressions of genes also change with age and contribute to the development of ARDs [12]. Thus, the identification of age-associated gene co-expressions will add an additional layer of capacity to understand aging, ARDs, and their connections.

Generally speaking, co-expressed genes are expected to be involved in the same functional processes [13]. Due to their biological importance, a lot of methods have been proposed to identify gene co-expressions and co-expression networks, most of which calculate simple statistical measures between the expressions of two genes across samples, such as the Pearson’s correlation, rank correlation, the Euclidean distance, and angle between expression vectors [14]. Other algorithms like weighted gene co-expression network analysis (WGCNA) transform the simple Pearson correlation or Spearman correlation of a pair of genes into topological overlap information to incorporate neighbouring genes [15]. There are also methods to infer gene co-expression based on linear regression models [16] or tree based methods [17]. After gene co-expressions have been quantified, clustering algorithms are useful for identifying groups of genes with similar expression profiles. For example, Eisen et al. used the hierarchical clustering to group co-expressed genes, and found that genes within a group were functionally related [13]. Smet et al. adopted K-means method to cluster gene expressions [18]. Other gene clustering methods include self-organizing map [19], graph based methods [20], and so on.

However, gene co-expression might be a consequence of various mechanisms. It is very difficult to determine those resulted from a single biological process like aging via gene expression matrix. Fortunately, Li proposed a concept called liquid association (LA) to describe how the dynamics of the association between two variables is mediated by a third one [21]. As an example, he studied the association of gene pair with an LA-scouting gene Z as a surrogate for the intrinsic cellular state that may affect the LA activity. There are two classical approaches using the liquid association: (i) for a given gene pair, screen the genome to detect the LA-scouting genes; (ii) for a given gene, screen the genome to find the liquid association pairs (LAPs). In addition, the mathematical definition of LA was given and a simplified formula for calculation was proven under some conditions. After the proposal of LA, many literatures have demonstrated the validity of the method in biology. For example, liquid association has been applied to find candidate genes for multiple sclerosis [22]. It has also been verified to be useful in performing dimension reduction in survival analysis [23]. Li and Yuan combined drug activity and gene expression profiles together and employed LA to find potential drug target genes [24].

In this study, we proposed an LA based method called Age Associated Gene Co-expression Identifier (AAGCI) to identify co-expressed genes whose association dynamics is correlated with age. Considering that the aging process has strong effects on many specific biological processes like mitochondria functions and inflammation pathways, we also restricted our methods into gene sets associated with terms defined by Gene ontology (GO) [25] and Kyoto Encyclopedia of Genes and Genomes (KEGG) [26]. We then compared our results with general gene co-expressions inferred by the Pearson correlation, cross-checked genes involved in age-associated co-expressions with known aging genes, and performed function enrichment analysis. Finally, we prioritized anti-aging drugs based on a simple enrichment analysis between aging co-expressed genes and drug perturbation signatures from a recent study [27].

## RESULTS

### AAGCI: an LA based model to identify aging associated gene co-expressions

**Figure 1:**
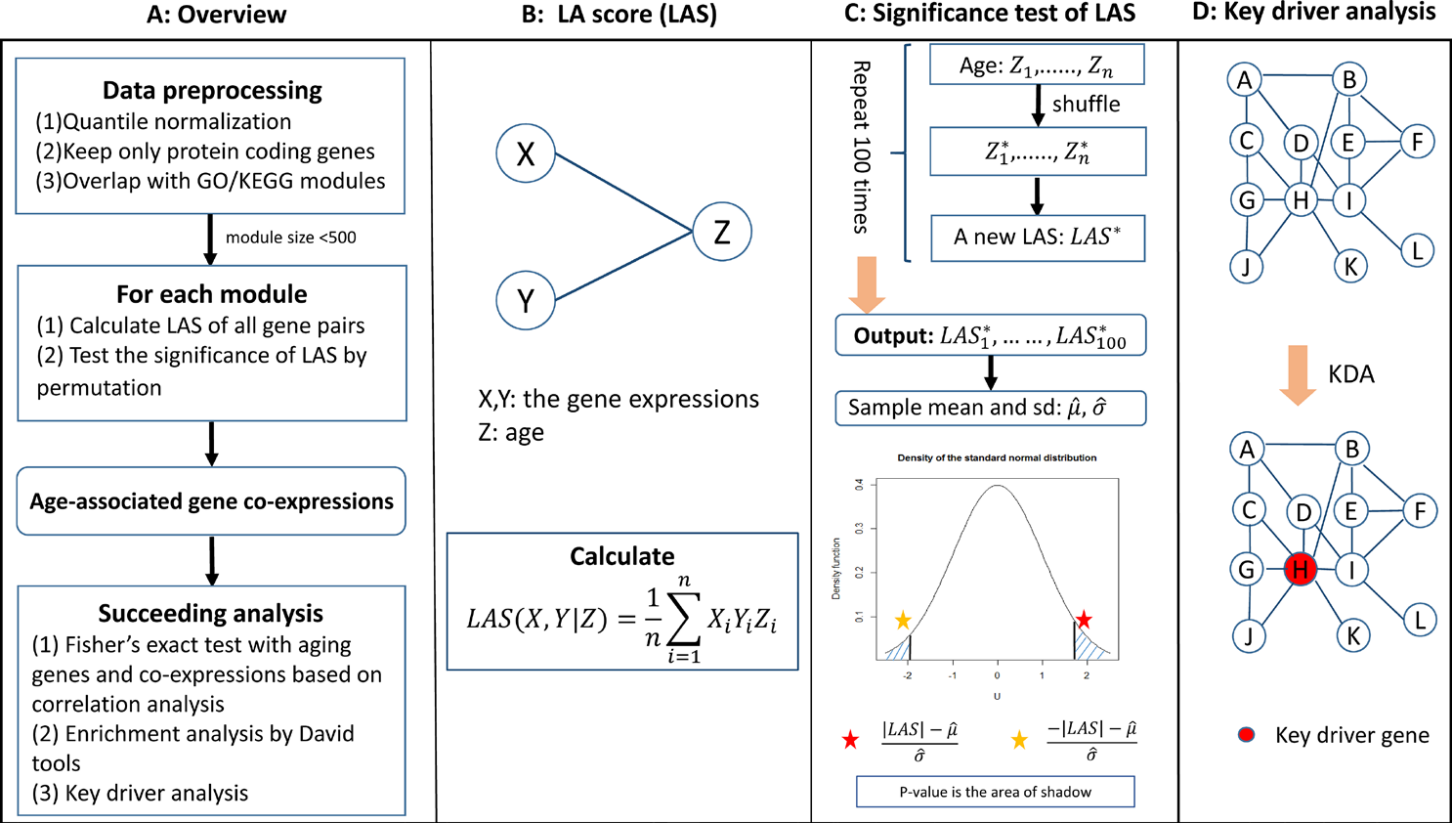
(**A**–**D**) An overview of the AAGCI algorithm.

We presented an overview of the AAGCI framework in Figure 1. Specifically, we first restricted our study into protein-coding genes and normalized the age and raw expression profiles across samples. We then overlapped the remaining genes with Gene Otology (GO) terms or KEGG pathways to form functional modules. For each module, we calculated liquid association score (LAS) dependent on age for each gene pair and tested its significance by a permutation study, which was further adjusted by the Benjamini-Hochberg method [28] to control the false discovery rate (FDR) for multiple testing. The gene pairs with FDR less than or equal to 0.1 were considered as age co-expressed and their key drivers were inferred by key driver analysis [2] on the protein-protein interaction subnetwork defined by the module genes. In addition, a gene involved in any liquid associated gene pair in any module is defined to be an age co-expressed gene. For a clear view, we also illustrated the calculation of LA scores, the assessment of LA significance by permutation analysis, and key driver analysis in Figure 1B–1D respectively. The readers were referred to Materials and Methods section for more details on each step.

### Identification of tissue specific age-associated gene co-expressions using the GTEx data

We applied AAGCI into the Genotype-Tissue expression (GTEx) pilot phase data to identify gene co-expression varied with age. The GTEx pilot phase (v3) provided 1,641 whole transcriptome profiles in more than 40 tissues from nearly two hundred post-mortem human donors [29]. Nine tissues had sample sizes of greater than 80, namely, adipose subcutaneous (adipose), artery tibial (artery), heart left ventricle (heart), lung, muscle skeletal (muscle), nerve tibial (nerve), skin sun exposed lower leg (skin), thyroid, and whole blood respectively. We considered these nine tissues in our study. We also obtained the GO terms and KEGG pathways using R package ‘org.Hs.eg.db’ and ‘KEGG.db’ (on June 13, 2016). To avoid too general functions, we only used GO terms and KEGG pathways with less than 500 genes, resulting in 12199 GO terms and 279 KEGG pathways.

It was found that some gene pairs were identified as significantly liquid associated in multiple GO terms or KEGG pathways. We listed in Table 1 the top 20 most frequently occurring liquid associated gene pairs in GO terms for adipose. The details of LA pairs of all tissues based on GO terms and KEGG pathways were shown in Supplementary Table 1. We are fully aware that GO terms and KEGG pathways might be overlapped, however it may not be a critical issue since our objective is to identify age-associated gene co-expressions at specific GO terms or KEGG pathways. Besides, it was suggested that the overlapping GO terms will not change the results a lot in a few network studies [2, 30].

**Table 1:** Top 20 most frequent age-associated gene co-expressions for modules defined by GO terms in adipose

Gene 1	Gene 2	Occurrence^*^	Gene 1	Gene 2	Occurrence
*PTPN6*	*STAT5A*	37	*IGF2*	*PPP1R3F*	19
*CITED2*	*WT1*	34	*STAT5A*	*TBX21*	19
*CARD11*	*TLR4*	28	*ABR*	*ADORA2B*	18
*IKZF1*	*STAT5A*	28	*STAT6*	*TBX21*	18
*CARD11*	*TNFRSF21*	24	*GTSE1*	*UBA52*	17
*ADORA2B*	*UNC13D*	20	*POLD3*	*RPA3*	17
*STAT5A*	*SYK*	20	*PSMC3*	*TP53*	17
*SYK*	*UNC13D*	20	*PSME1*	*UBA52*	17
*CD3E*	*TNFRSF21*	19	*GTSE1*	*PSMB4*	16
*CD86*	*IL13*	19	*POLD1*	*RFC4*	16

^*^Occurrence counts the number of network modules (by GO terms), in which Gene1 and Gene2 are significantly age co-expressed

**Figure 2:**
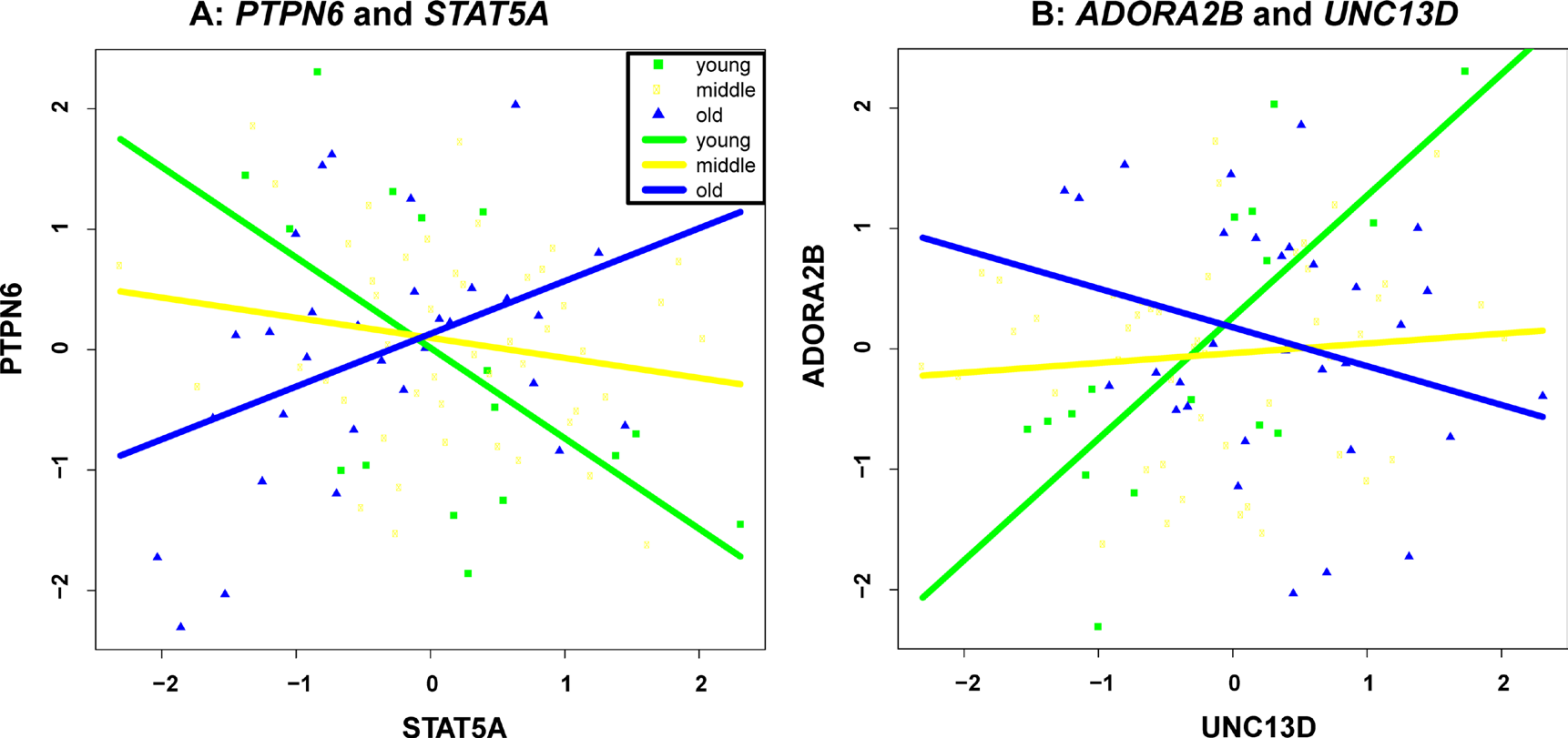
Group-based gene-gene correlation of “*PTPN6, STAT5A*” (**A**) and “*ADORA2B, UNC13D*” (**B**).

To ensure that the co-expressions of the identified gene pairs in Table 1 are indeed associated with age, we separated the samples into 3 groups, namely young (age < = 35), middle (age between 35 and 55), and old (age > = 55) according to their age, and checked gene-gene correlation at the 3 groups (see Supplementary Figure 1). As two examples, we also illustrated in Figure 2 the group-based gene-gene correlation of the top two pairs, i.e., “*PTPN6*, *STAT5A*” and “*ADORA2B*, *UNC13D*”. It is clear that the co-expressions of “*PTPN6*, *STAT5A*” gradually change from negative to positive as sample age increases. In contrast, the co-expressions of “*ADORA2B*, *UNC13D*” decrease with the increase of age.

Interestingly, we also find that most genes in Table 1 have been reported to be associated with aging or age-related biological processes. For example, *PTPN6,* also known as *SHP-1* or tyrosine-protein phosphatase non-receptor type 6, regulates a variety of cellular processes like cell growth, differentiation and oncogenic transformation [31]. It was inferred to be age-associated in human [4] and mouse [32]. *STAT5A*, is a transcription factor mediating cellular responses to growth factors and promoting transcription of genes associated with proliferation, differentiation, and survival of cancer cells [33]. *STAT5A* was selected as a potential human aging gene in GenAge due to its close relationships with known aging genes including *GHR*, *GH1*, and *IGF1* (http://genomics.senescence.info/genes/entry.php?hgnc = Stat5a). In addition, *PTPN6* interacts with *JAK2*, which is very important in the function of *STAT5A* [34]. Similarly*, CITED2* and *WT1*, the second pair in the list, have been shown to interact with each other to stimulate expression of the nuclear hormone receptor Sf-1 (Nr5a1) in the AGP to ensure adrenal development [35]. The co-function of these gene pairs in aging-related cellular or biological processes indicates that they might be indeed age co-expressed. Besides the top two pairs, a few genes in the list like *TP53* and *IGF2* are known aging genes in GenAge [36].

### Functional annotation of age co-expressed genes leads to a large collection of biological processes

**Figure 3:**
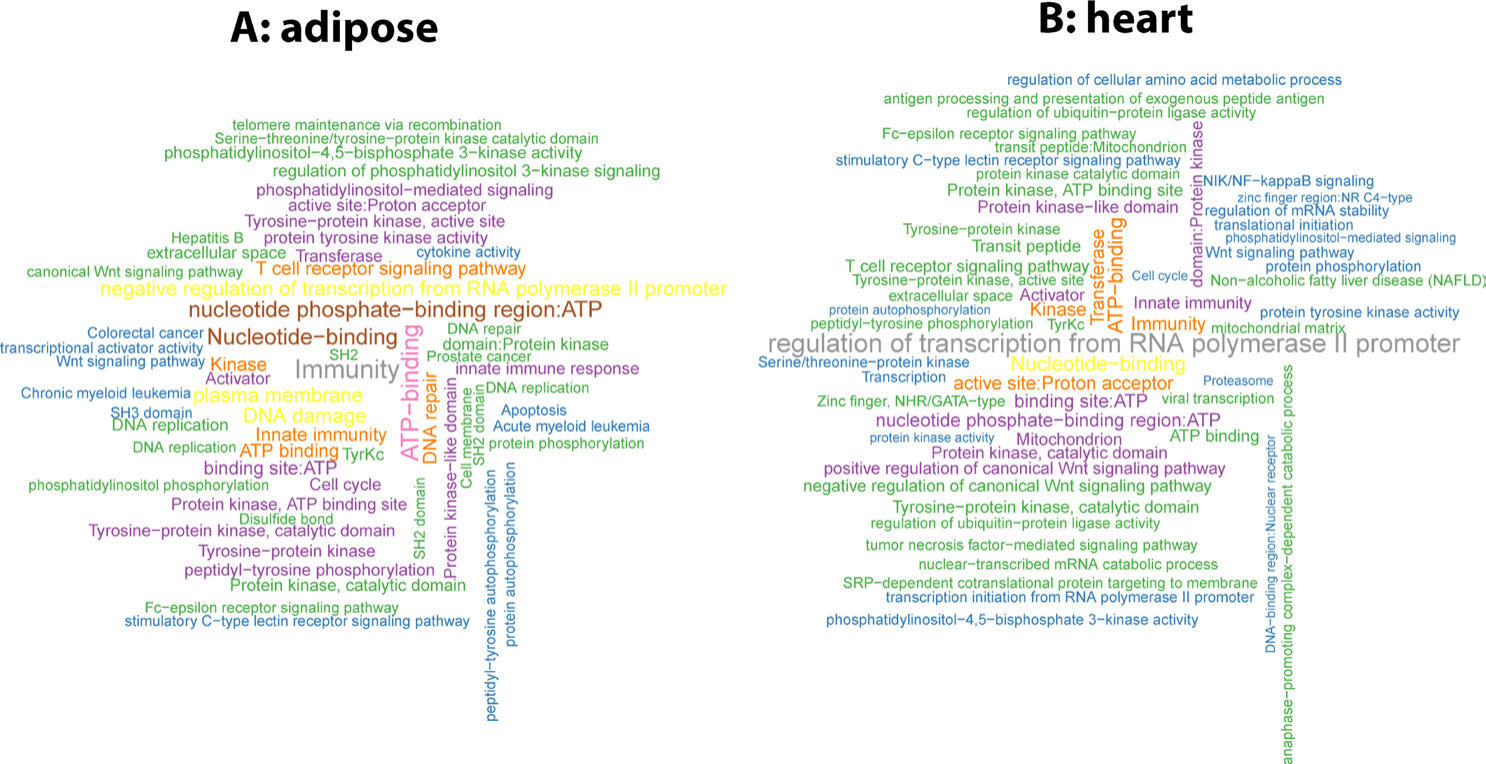
Word-cloud plots of the functional annotation of age co-expressed genes in two tissues (**A**) adipose and (**B**) heart.

To present an overview of age co-expressed genes, we annotated them by the David tools (version 6.8). As two representative examples, we plotted in Figure 3A and 3B the word-cloud maps of the enrichment for tissues adipose and heart respectively. We also showed a few top representative annotations for adipose in Table 2 and provided the enrichment results for all 9 tissues in Supplementary Table 3. As can be seen from Table 2, the term “immunity” is most enriched in adipose with FDR 1.32E-48. It is widely known that the aging process deteriorates the immune system and the immune system in turn affects longevity and age-related diseases [37]. Other top terms in the list such as “ATP-binding”, “DNA damage”, and “DNA repair” are also well known to be critical in the aging process.

**Table 2:** Functional enrichment of age co-expressed genes in adipose

Module	*p*-value	FDR
Immunity	8.96E-50	1.32E-48
ATP-binding	1.45E-39	2.13E-38
Nucleotide-binding	1.69E-36	2.49E-35
nucleotide phosphate-binding region:ATP	1.02E-34	1.96E-33
GO:0005886 plasma membrane	9.65E-32	1.52E-30
DNA damage	1.14E-28	1.68E-27
GO:0000122 negative regulation of transcription from RNA polymerase II promoter	9.09E-29	1.81E-27
Innate immunity	3.75E-27	5.53E-26
GO:0050852 T cell receptor signaling pathway	9.88E-27	1.97E-25
Kinase	1.09E-25	1.60E-24
GO:0005524 ATP binding	2.38E-25	4.09E-24
DNA repair	7.78E-25	1.15E-23
binding site:ATP	1.23E-22	2.37E-21
Cell cycle	1.55E-21	2.29E-20
Transferase	2.90E-20	4.27E-19
IPR011009:Protein kinase-like domain	1.12E-19	2.01E-18
IPR017441:Protein kinase, ATP binding site	1.17E-19	2.10E-18
Activator	2.01E-19	2.96E-18

**Figure 4:**
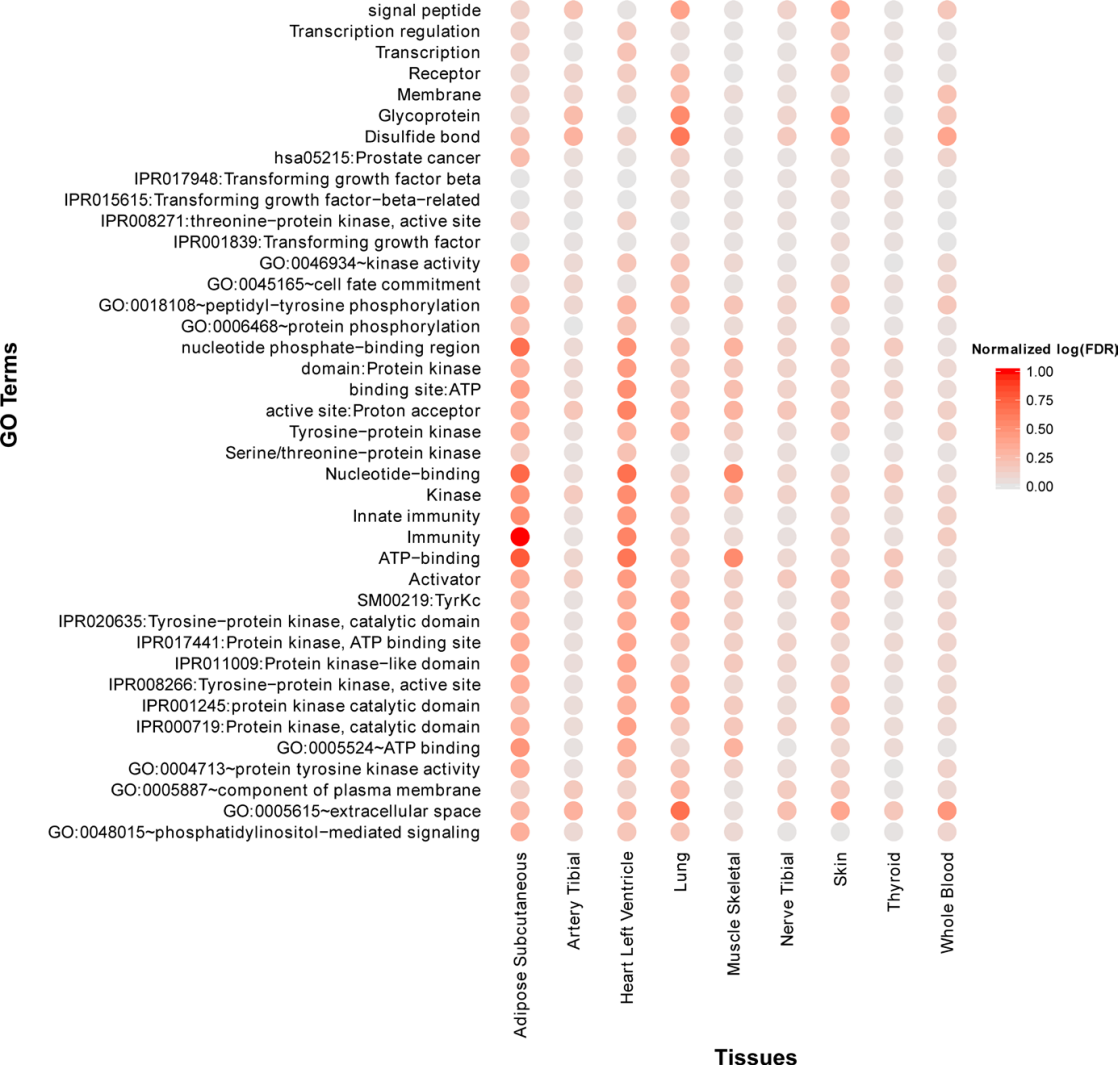
Top 40 frequently enriched GO terms and KEGG pathways of age co-expressed genes across multiple tissues Normalized *log(FDR)* is defined as [*max(log(FDR))-log(FDR)]/[max(log(FDR))-min(log(FDR))*]. A large “normalized log(*FDR*) ” indicates a more significantly enriched item.

By examining the function annotations across all tissues (see Supplementary Table 3), we identified a few common terms (Figure 4). For example, the terms “Immunity” and “Innate immunity” are significantly enriched across all tissues, which recaptures the critical roles of immunity in aging process. Other terms like “IPR017441:Protein kinase, ATP binding site” are also enriched in most tissues. In addition, we observed that many cancer pathways are significantly enriched in multiple tissues, for example, “hsa05217:Basal cell carcinoma” (adipose, artery tibial, lung, nerve tibial, skin, thyroid), “hsa05218:Melanoma” (adipose, lung, skin, thyroid, whole blood), “hsa05213:Endometrial cancer” (adipose, lung, thyroid, whole blood) and “hsa05215:Prostate cancer” (adipose, lung, skin). It is no surprise since aging and cancer share many common biology such as genomic instability, immune response, and autophagy [38].

### Age co-expressed genes inferred by AAGCI significantly overlap with known aging genes

Given the age-associated gene co-expressions, one natural question is how they are related to known aging genes. To answer this question, we compared age co-expressed genes with GenAge genes. GenAge is a benchmark dataset of aging genes, in which 305 aging genes were curated from over 1000 references (on 12-30-2016) [36]. We listed the enrichment results in Table 3. As can be seen, the ratios of age co-expressed GenAge genes vary from 15.41% to 45.25% across different tissues. We also implemented the one-sided Fisher’s exact test to assess the overlapping significance between the two sets. As a result, the *p*-values for the tests range from 5.91E-14 (skin) to 5.21E-51 (adipose) for all 9 tissues, indicating a tissue-wide correlation between age-associated genes and co-expressions.

**Table 3:** Overlap between age co-expressed genes and GenAge genes

Tissue	^#^Age co-expressed genes	#GenAge genes	Background Genes	#Overlap genes	Ratio^*^	*P*-value#
adipose	2563	305	16516	155	50.82	5.21E-51
Artery	821	305	16096	56	18.36	4.45E-18
heart	2490	305	15721	137	44.92	8.02E-37
lung	1157	305	16853	80	26.23	3.68E-27
muscle	2025	305	15928	117	38.36	3.60E-33
nerve	769	305	16557	56	18.36	3.68E-20
skin	774	305	16733	47	15.41	5.91E-14
thyroid	1260	305	16737	69	22.62	1.09E-17
whole blood	1035	305	16025	69	22.62	1.09E-17

^*^Ratio is calculated as the number of overlap genes divided by that of the GenAge genes. #P-value is calculated by the one-sided Fisher’s exact test.

### Age-associated gene co-expressions are different from general gene co-expressions

One of the conventional methods to infer gene co-expressions is through the Pearson correlation coefficient (PCC) [14]. We compared the age associated gene co-expressions with strongly correlated gene pairs detected by PCC. For a fair comparison, the co-expression study via PCC was conducted in the same process of identifying age-associated gene co-expressions, i.e., highly correlated gene pairs were detected in GO or KEGG modules. The Benjamini-Hochberg method [28] was used to control FDR at level 0.1. Then in each GO term or KEGG pathway, the one-sided Fisher’s exact test was carried out to test whether the gene co-expression studies through LA and PCC were consistent. Finally, the Benjamini-Hochberg method were employed to correct multiple testing across all GO and KEGG terms and adjusted *p*-values were reported in Supplementary Table 2. As can be seen, age-associated LA gene pairs and PCC gene pairs were significantly overlapped only in 13 (over an overall of 12478) modules for heart, in 1 module for adipose and 0 module for the other 7 tissues. Though small sample size and sequencing quality might contribute to the inconsistency, the results generally suggest that age-associated gene co-expressions are different from gene co-expressions.

### Identification of key driver genes

**Figure 5:**
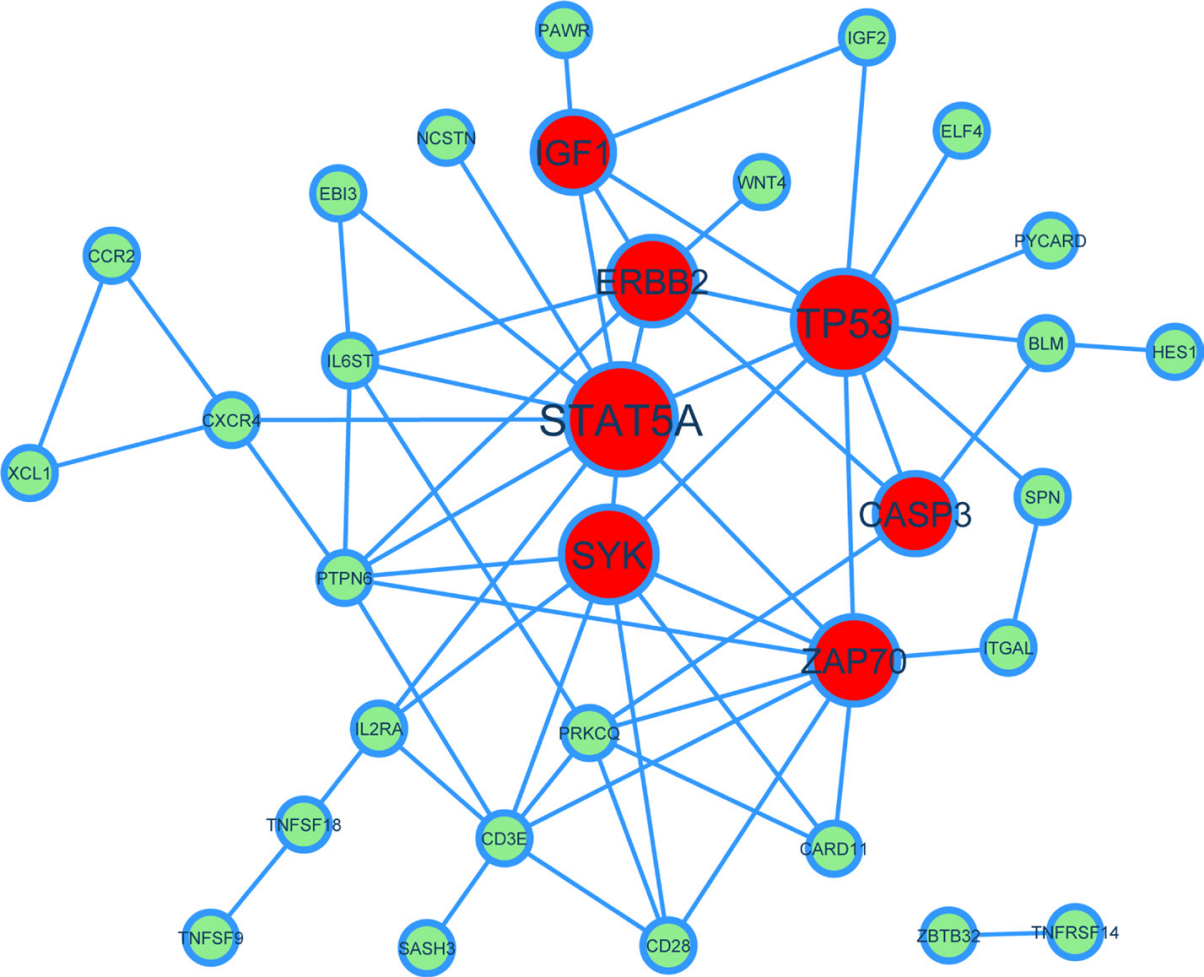
A network view of key drivers of aging co-expressed genes for the module GO:0042098 “T cell proliferation” In the PPI network. Red nodes represent key drivers and light green nodes represent other genes. Node size represents the rank of key drivers.

For the modules where aging co-expressed genes were recognized, it is important to identify their key driver (KD) genes in the module network, which could be drug targets. We applied a similar approach with Yang et al. [39], in which we used protein-protein interaction (PPI) network defined by the HPRD database (http://www.hprd.org) as the reference network (see Materials and Methods for details). We used module GO:0042098 “T cell proliferation” as an example to illustrate. The network of the KDs and their connectivity was shown in Figure 5 via Cytoscape, in which KDs were drawn in red and other genes in green. As can be seen, there are 7 key driver genes including *IGF1*, *ERBB2*, *TP53*, *STAT5A*, *CASP3*, *SYK*, and *ZAP70*, among which *IGF1*, *ERBB2*, *TP53* and *STAT5A* are known GenAge genes [36]. *IGF1* (insulin-like growth factor 1 receptor) has been shown to be associated with lifespans of fruit flies and nematodes [40]. *TP53* is probably the most studied tumor suppressors and aging genes, which is also a key regulator of the DNA damage responses [41]. Moreover, though *CASP3*, *SYK*, and *ZAP70* are not GenAge genes, there are evidences for them to be related with aging and ARDs. For example, *CASP3* is important in the development of Alzheimer’s disease in senescent brains [42]. As a conclusion, the key driver analysis on aging co-expressed genes is capable of prioritizing critical aging or ARD genes for further experimental validation.

### Prioritize anti-aging drugs using aging associated LA genes

Finally, we utilized the genes involved in age-associated gene co-expressions to prioritize anti-aging drugs based on drug perturbation signatures from a recent study [27]. For each aging co-expressed gene, we first calculated the Pearson correlation between its expression profile and age (across samples), and carried out a significance test (two-sided *t*-test) for correlation coefficients with *p*-value adjusted by Benjamini-Hochberg method [28]. The aging co-expressed genes with false discovery rate under 0.1 were kept. In addition, an aging co-expressed gene is called up-regulated (or elevated) with age if the correlation coefficient is positive, otherwise it is called down-regulated (or repressed). In [27], Wang et al. manually curated lists of 4,295 single-drug perturbations and 8,620 single-gene perturbations from Gene Expression Omnibus. Specifically, we try to infer either drug or gene perturbations that could possibly reverse the aging signatures (i.e., perturbations that up-regulate those repressed aging gene expressions or down-regulate those elevated aging gene expressions). In practice, we performed the enrichment analysis between the repressed (elevated) aging co-expressed genes and up-regulated (down-regulated) drug perturbation genes using the Fisher’s exact test. A similar analysis is also performed on gene perturbation signatures. The drugs (genes) were then ranked based on the *p*-value for the test. We listed the results for all tissues in Supplementary Table 4 (except for thyroid since no significant up- or down-regulated aging co-expressed genes could be found).

Since metformin, a type 2 diabetes medicine, is one of the best studied and promising anti-aging drugs [43, 44], we listed our prediction results for this drug across multiple tissues in Table 4. We can see that metformin locates at top 10% out of the 4295 drugs at several tissues. Interestingly, metformin is ranked high for both up-regulated (*p* = 0.0022, ranked at 198^th^) and down-regulated (*p* = 0.0063, ranked at 416^th^) aging co-expressed genes in lung. This is consistent with a few literatures that metformin possesses protective effect on lung cancer [45–47]. Besides lung, metformin also ranks 63th in muscle, 130^th^ in nerve, and 294^th^ in adipose, suggesting its possible anti-aging effect on whole human body.

**Table 4:** Predicting the anti-aging effect of metformin across tissues

Tissue	Up-regulated		Tissue	Down-regulated	
	Rank^*^	*P* value		Rank	*P* value
lung	198	0.0022	muscle	63	0.0808
artery	1039	0.0540	nerve	130	0.0723
			lung	416	0.0063
			adipose	294	0.0001

^*^Rank is inferred based on *p*-values for all 4295 drugs with low *p*-value ranks first.

Besides metformin, we also prioritized a few other potential drugs to slow down or reverse aging and age-associated diseases. For example, insulin related drugs are listed at top for most tissues (see Supplementary Table 4). It is known that excess insulin is one of the main causes of accelerated aging, and thus drugs controlling insulin levels might have potential anti-aging effects. The reader are referred to Supplementary Table 4 for more prioritized drugs.

## DISCUSSION

Gene co-expression is an important mechanism as well as a strong evidence for genes to function corporately. There are a number of computational methods to infer gene co-expression including correlation based methods [14], WGCNA [15], regression-based methods [16], and so on. However, almost all existing methods focus on general gene co-expressions, while those results from specific mechanisms like aging are more or less ignored possibly due to data limitation and computational complexity. Recently, the GTEx project generated multiple tissue gene expression data for hundreds of post-mortem individuals with a wide range of age (20-70), providing a very good resource for aging study. Thus, we applied an LA-based method to infer age-associated gene co-expression into the GTEx pilot phase data on 9 human tissues. The LA-based method is capable of catching the dynamics of the association between two gene expressions mediated by aging.

We inferred thousands of age-associated gene co-expressions for different tissues and each tissue has different numbers of significant gene co-expressions. This result is consistent with our previous study on age-associated gene expressions [4]. The top genes involved in age-associated gene co-expressions are enriched in biological functions like immunity, ATP binding, DNA damage, and so on. We also prioritized a few anti-aging drugs based on a similar strategy to connectivity map [48]. It is worth noting that the drug signatures from CROWD suffer from false-positives and incompleteness, and thus the drugs predicted in this study might not be very accurate. Nevertheless, our study predicted a few known anti-aging drugs and a few meaningful candidates.

There are some limitations of our method. First of all, our method measures the absolute mean of the derivative of the covariance of two gene expressions given age, which in theory will cause false-negatives. For example, when the age-associated gene co-expression is positive for an individual from 20-50 and negative from 50-70, the absolute mean might be close to 0 and will be insignificant. Nonetheless, AAGCI is capable of capturing most age-associated gene co-expressions and presents meaningful results. Second, AAGCI does not account for the overlap in the GO and KEGGs. It is known that Gene Ontology has a hierarchical structure and thus some GO terms are highly overlapped. We will mine the effect of this factor to our method in the future. Third, we used only the overlapping of the drug signatures and age-associated co-expressed genes to infer anti-aging drugs. The simple method does not consider the weight of genes and also the intrinsic interaction (like PPI interactions) among genes. A more complex method involving this information is highly desirable. Fourth, there are many other co-regulation patterns like gene regulations and protein co-expressions, however we focus on age-associated gene co-expressions in this paper since the gene expression data is most accessible. Bayesian network is widely used to infer gene regulations, however it usually needs additional information like transcription factors and expression quantitative trait loci (eQTLs) to help determine the regulation direction.

Finally, it is worth mentioning that though we studied age-associated gene co-expressions in this study, the proposed method may have a few further applications. In principle, they could be used to study gene co-expressions dependent on any quantitative trait. For example, by replacing age to BMI, one can study the obesity association gene co-expressions. Similarly one can study drug sensitivity associated gene co-expressions from studies like Cancer Cell Line Encyclopedia (CCLE) [49] and Library of Integrated Network-based Cellular Signatures (LINCS, http://lincsproject.org/). Another interesting topic is to study the gene co-expressions associated with environmental factors (like smoking, drinking or microbes) and diseases. However, it is out of the scope of this study.

## MATERIALS AND METHODS

### Data source

*GTEx data*: GTEx data (v3, December 2012 release) provides expression levels of 41,298 genes in nine human tissues: adipose, artery, heart, lung, muscle, nerve, skin, thyroid, and whole blood. The sample size of each tissue ranges from 83 to 156. The detailed information on sample collection, RNA collection, RNA-Seq experiment, gene expression estimation, quality control, and gene expression normalization was provided elsewhere [29].

### Liquid association

The terminology “liquid association” (“liquid” as opposed to “solid”), was first proposed to conceptualize the internal change of association patterns for a pair of genes (X,Y) in response to constant changes with of cellular state variables [21]. Since the relevant cellular states are unknown, in the literatures the cellular state was often assumed to be associated with the expression of a certain gene Z, called as LA-scouting gene. Many other factors which influence the cellular state can take the place of the scouting gene Z. In this paper, we use “age” as the scouting variable to search the gene pairs that have the liquid association patterns.

The mathematical definition of liquid association was given based on the three random variables X, Y and Z [21]. It was assumed that X, Y and Z are random variables with mean 0 and variance 1. The liquid association score (LAS) of X, Y with respect to Z is defined by LA(X,Y│Z)=Eg' (Z), where g(Z)=E(XY|Z). Furthermore, if Z follows the standard normal distribution, the equivalent expression of LA has been proved that by using the celebrated Stein Lemma [50]. So the calculation can be dramatically simplified. Then when Z is standard normal, the LAS can be calculated by the formula,

$$LAS\left(X,Y|Z\right)=\frac{\sum_{i=1}^{n}X_{i}Y_{i}Z_{i}}{n}$$

where X_i_,Y_i_ denotes the expressions of the genes X and Y respectively for the i-th individual,Z_i_ represents for the scouting variable, and *n* is the sample size, i.e., the number of individuals (measurements).

### Data pre-processing including quantile normalization and filtration

In the pre-processing, we planned to do some adjustment by regression to the raw data. However, considering that the adjustment to the gene expressions by a regression model with some covariates like age, gender and etc., is possible to remove some useful information regarding the liquid association between genes, we finally chose to use the raw data for the normalization and filtration. In fact, all the gene expression profiles and age were inverse-normal transformed to the standard normal distribution to satisfy the distribution assumption in the liquid association definition [21]. Furthermore, taking into account that only the protein coding genes have biological meaning in the protein synthesis and the subsequent biological functions, after normalization we then filtered the genes to get the overlap with 20069 protein coding genes downloaded from HGNC (http://www.genenames.org/cgi-bin/statistics). Finally, 15721 to 16853 genes were reserved for the 9 tissues in the further analysis.

### Search for liquid association based on GO and KEGG functional modules

To avoid the huge burden of screening LA pairs (LAPs) across the whole gene expression data, and due to the phenomenon of gene functional modulization, we focused on LAPs screening in functional modules defined by Gene Ontology (GO) terms and Kyoto Encyclopedia of Genes and Genomes (KEGG). Specifically, for every tissue, each module of GO and KEGG was taken to overlap with the processed data and only the modules containing less than 500 genes were considered. The intra-module search for LAPs were implemented. The genes within each GO term and KEGG pathway were obtained using R package “org.Hs.eg.db” and “KEGG.db”, respectively on June 13, 2016.

### Permutation test and false discovery rate (FDR)

In order to detect the gene pairs with significant liquid association score (LAS), we used the permutation test as suggested by [21]. Specifically, the observations of the age were shuffled, and which consequently generated a corresponding new LAS value. Due to the enormous amount of genes and the huge computational cost, *100* permutations were implemented. We denoted the new LASs generated from permutations by $\left\{{LAS}_{1}^{*}\left(X,Y\right),\ldots ,{LAS}_{100}^{*}\left[X,Y\right]\right\}$, which could be regarded as a random copy of *LAS(X,Y)*. Then the *p*-value of the significance test was the probability of |LAS*| greater than |LAS|. However, to avoid the rough *p*-value with the magnitude of 10^–2^, we did not compared *LAS(X,Y)* with the empirical distribution of the LAS* values. Instead, we assumed that the distribution of *LAS(X,Y)* is normal with mean and standard deviation σ. The estimates $\hat{\mu }$ and $\hat{\sigma }$ were calculated by the values of $\left\{{LAS}_{1}^{*}\left(X,Y\right),\ldots ,{LAS}_{100}^{*}\left[X,Y\right]\right\}$. Consequently, by the cumulative distribution function (CDF) of the standard normal distribution, the *p*-value could be expressed as,

$$p=1-\phi \left(\frac{\left|\mathrm{LAS}\left(X,Y\right)\right|-\mu }{\sigma }\right)+\phi \left(\frac{-\left|\mathrm{LAS}\left(X,Y\right)\right|-\mu }{\sigma }\right)$$

where ϕ (.) refers to the CDF of the standard normal distribution

Naturally, the gene pairs in the same module lead to a multiple testing problem. To control the false discovery rate (FDR), Benjamini-Hochberg method [41] was applied to adjust the *p*-values of the test problem (T1) for all gene pairs across the same module. We took the level of false rate as 0.10. The gene pairs with adjusted *p*-value not greater than 0.10 were finally considered as LAPs.

### Functional enrichment analysis

We carried out the functional enrichment analysis on the liquid associated genes for each tissue separately by David tools (http://david.abcc.ncifcrf.gov/summary.jsp). FDR score was used to control the false discovery rate and a gene set was considered to be significant if the FDR score is less than or equal to 0.10.

### Test the consistency of liquid associated genes and aging genes

We downloaded the list of 305 aging genes (on 12-30-2016) from the database GenAge (http://genomics.senescence.info/genes/) [36]. The one-sided Fisher’s exact test was adopted to check the consistency of aging genes and liquid associated genes. The *p*-value not greater than 0.05 can support that liquid associated genes are consistent with aging genes. The test was carried out for all tissues.

### Key driver analysis

The LA genes identified in the same GO or KEGG module was treated as a gene set. The protein-protein interaction (PPI) network was obtained from the HPRD database (http://www.hprd.org). We carried out key driver (KD) analysis to search the key drivers of the gene set, with the PPI network integrated. The KD analysis aimed to find the important genes for a gene set based on a given network. A gene whose neighbour genes in the network are significantly enriched for genes in the gene set consisting of LA genes is defined as a KD. We mapped the gene set into the PPI network. For each gene in the PPI network, we retrieved its directly connected genes (1st layer neighbour genes) to form a neighbouring subnetwork of the gene. Then, the Fisher’s exact test was used to evaluate the enrichment of the subnetwork genes. The genes in the PPI network with the neighbouring subnetworks which reached FDR adjusted *p*-value not greater than 0.05 were reported as KDs. The Cytoscape (http://www.cytoscape.org/) was adopted to draw the plots for the KDs and their connectivity in the PPI network.

### Data access

The GTEx genotype and gene expression data were downloaded from dbGap under dbGaP Study Accession number phs000424.v3.p1.

## SUPPLEMENTARY MATERIALS FIGURE AND TABLES










