# Supplementary material for: Identification of human age-associated gene co-expressions in functional modules using liquid association
Source: Oncotarget. 2017 Dec 8;9(1):1063–74. doi: 10.18632/oncotarget.23148 (PMC5787419; doi:10.18632/oncotarget.23148)
Supplement: Supplementary file 1 [file oncotarget-09-1063-s001.pdf]

Identification of human age-associated gene co-expressions in functional modules using liquid association

SUPPLEMENTARY MATERIALS

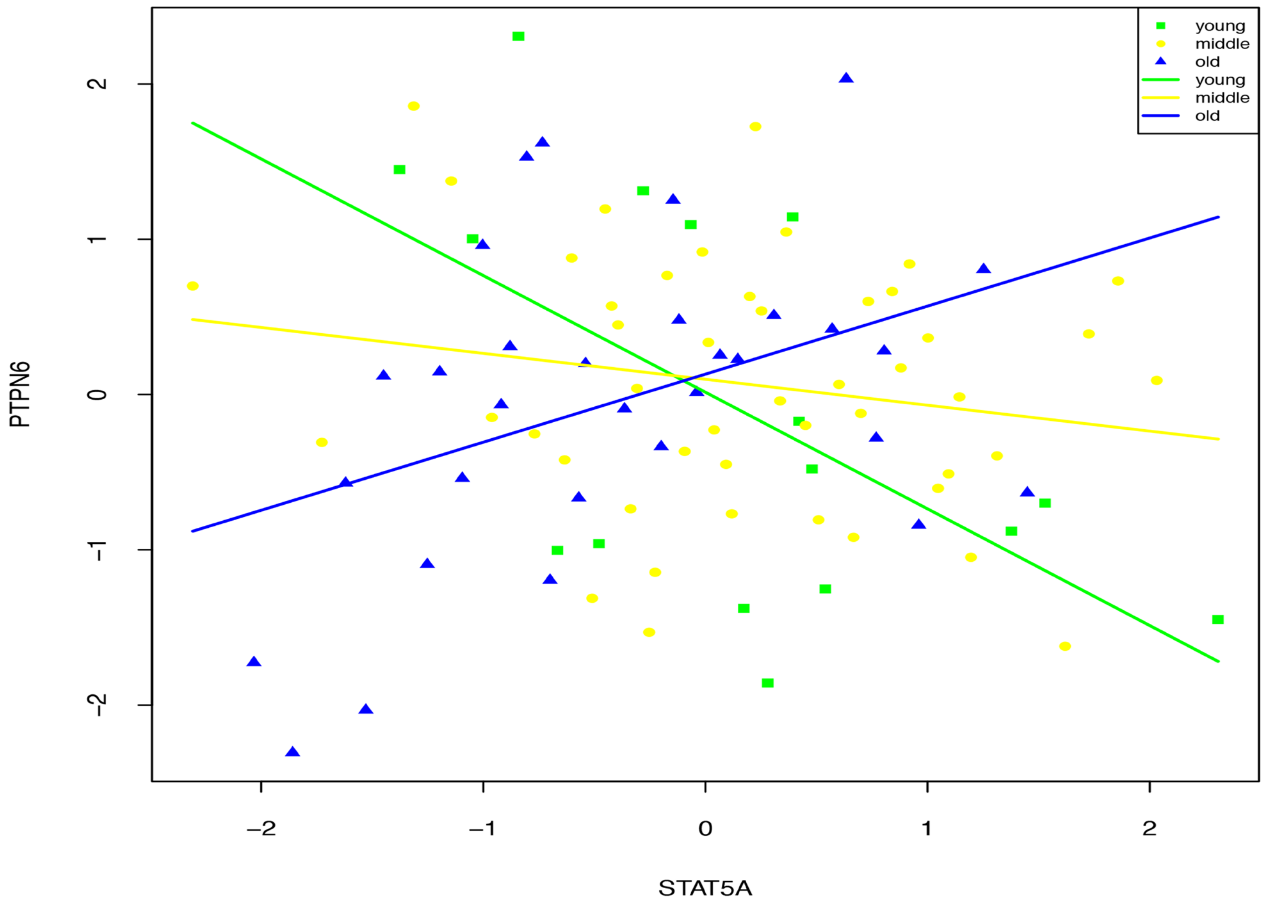

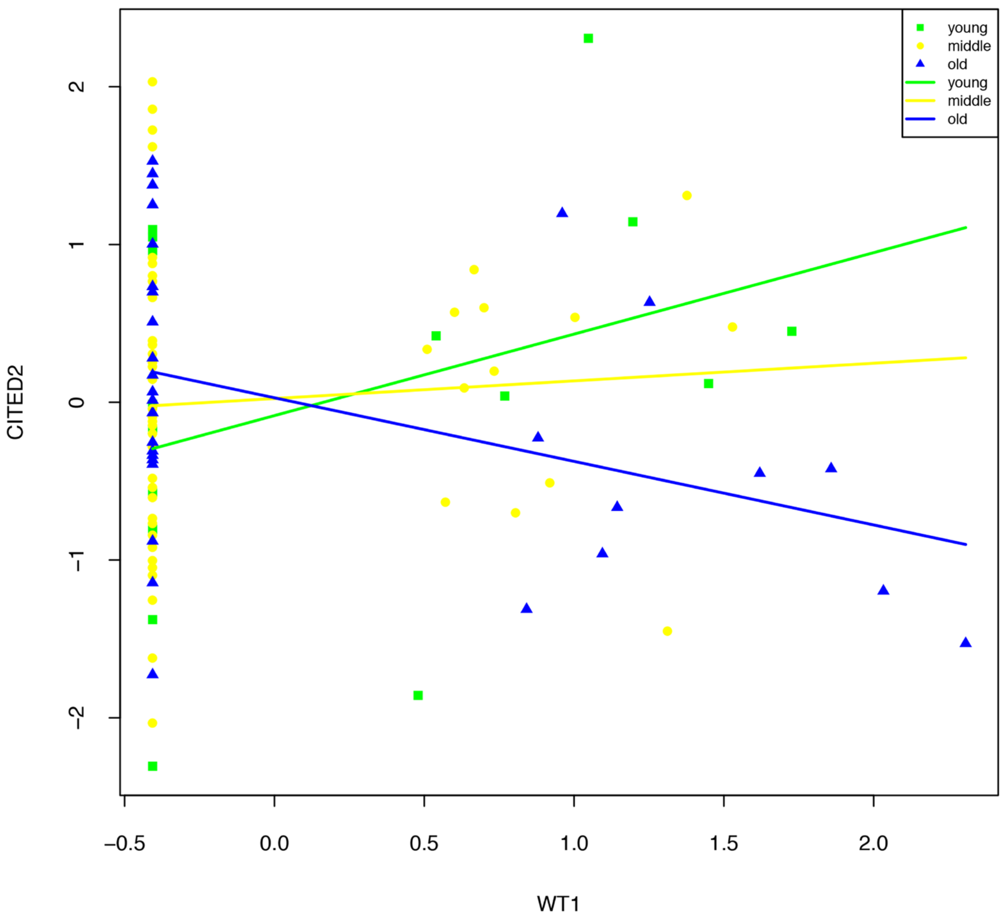
young
middle
old
young
middle
old
CITED2
WT1
−0.5
0.0
0.5
1.0
1.5
2.0
−2
−1
0
1
2

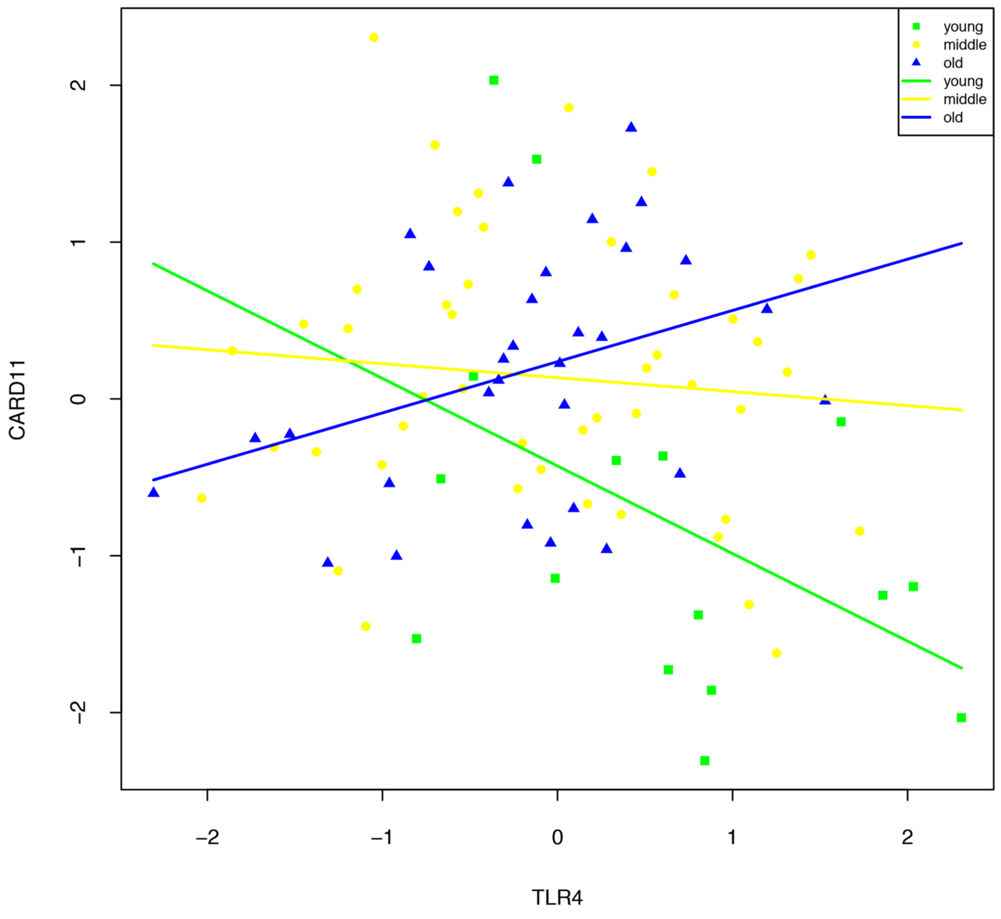
young
middle
old
young
middle
old
CARD11
TLR4
−2
−1
0
1
2

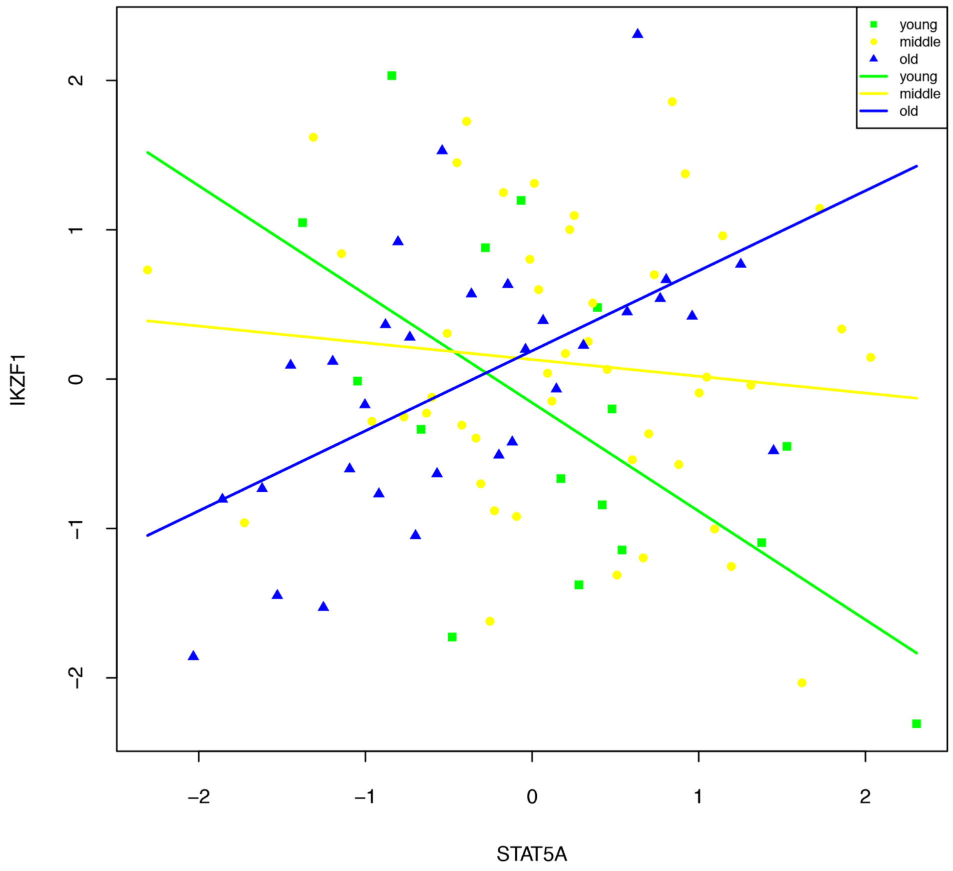

young
middle
old
young
middle
old
IKZF1
STAT5A
−2
−1
0
1
2

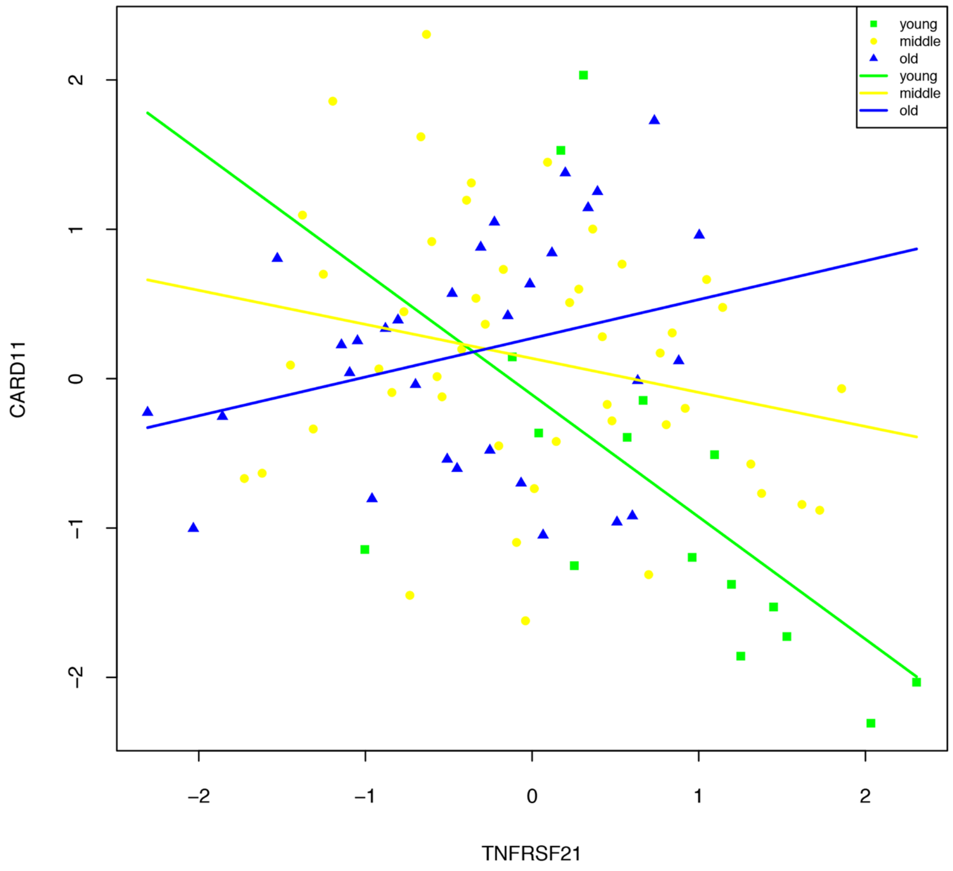

young
middle
old
young
middle
old
CARD11
TNFRSF21
−2
−1
0
1
2

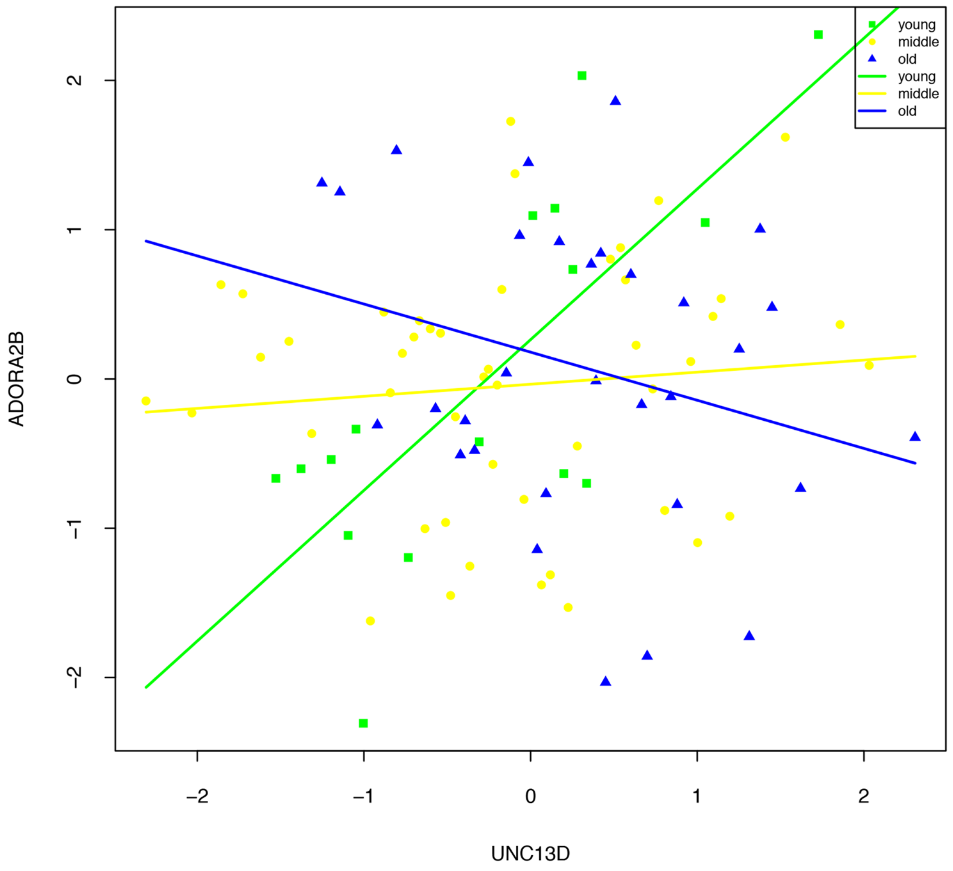
young
middle
old
young
middle
old
ADORA2B
UNC13D
−2
−1
0
1
2

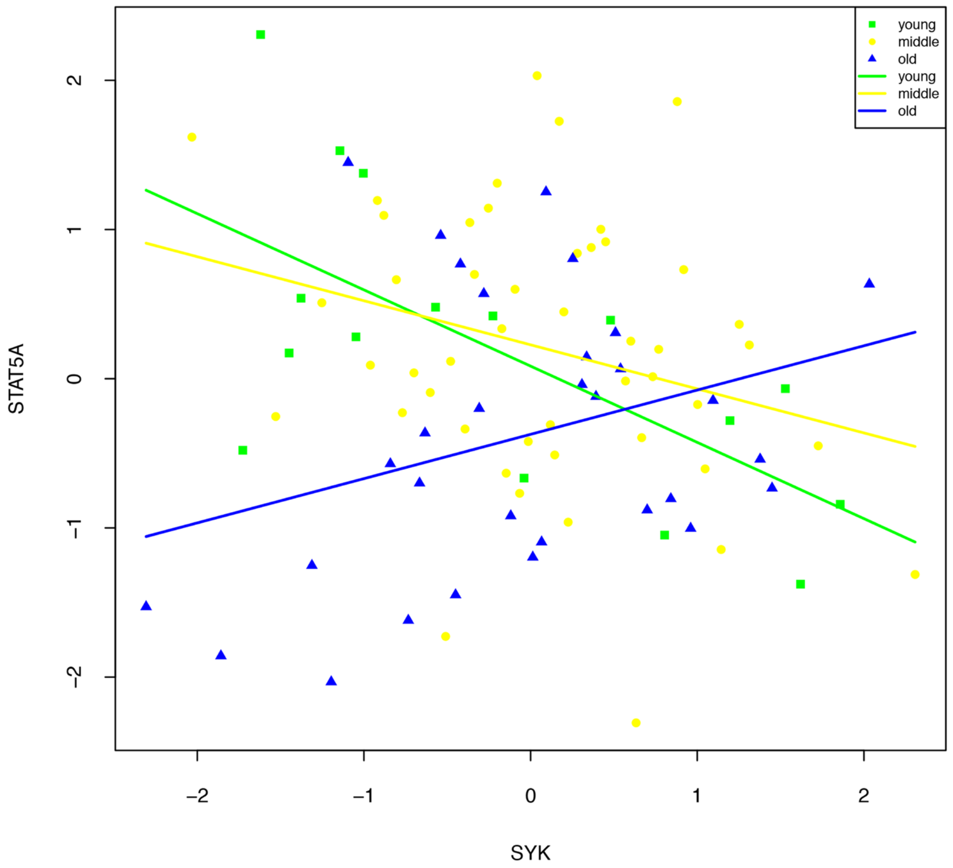
young
middle
old
young
middle
old
STAT5A
SYK
−2
−1
0
1
2

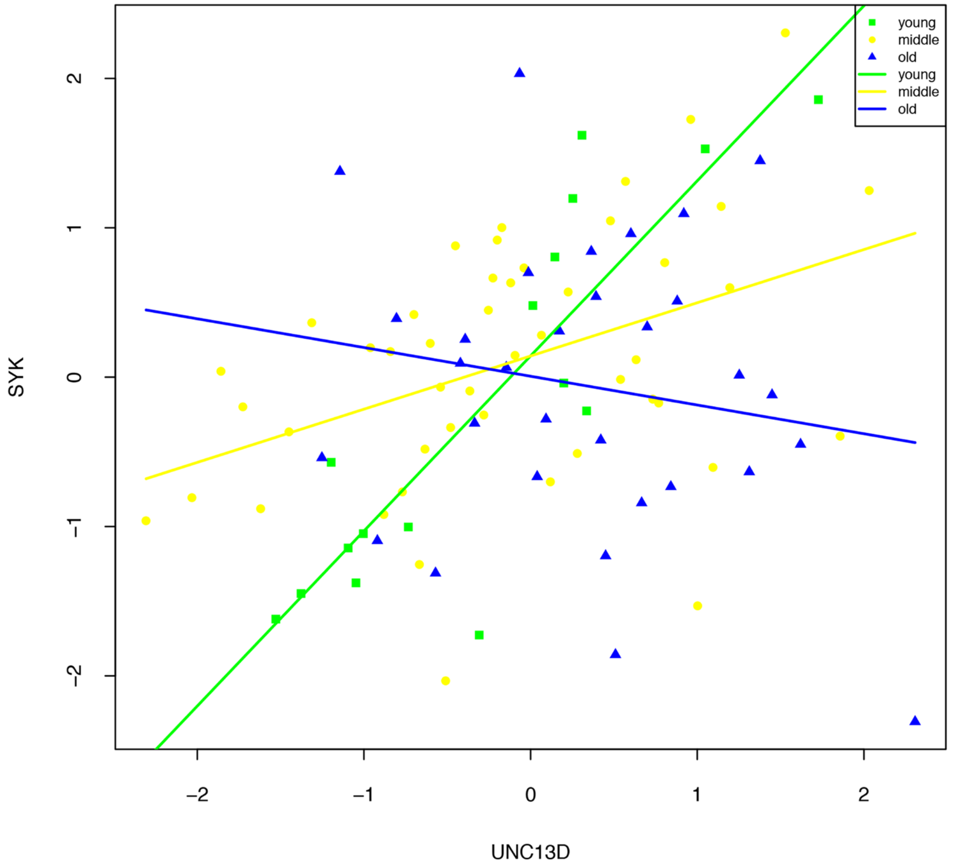

young
middle
old
young
middle
old
SYK
UNC13D
−2
−1
0
1
2

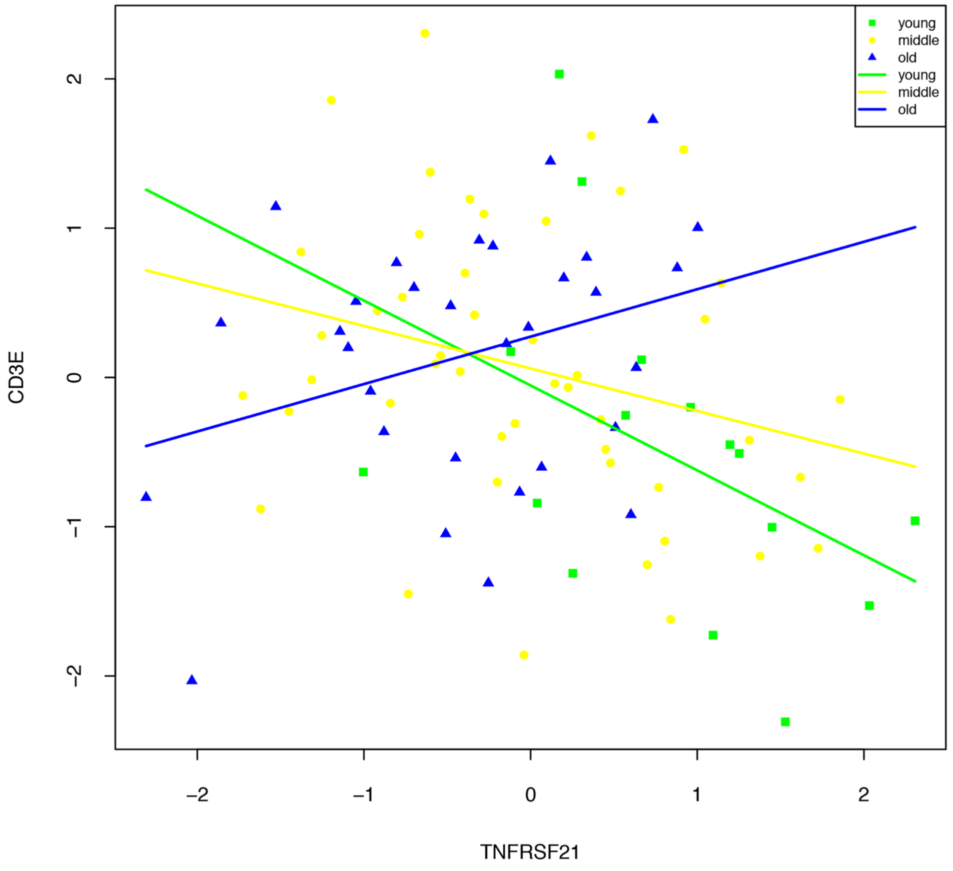

young
middle
old
young
middle
old
CD3E
TNFRSF21
−2
−1
0
1
2

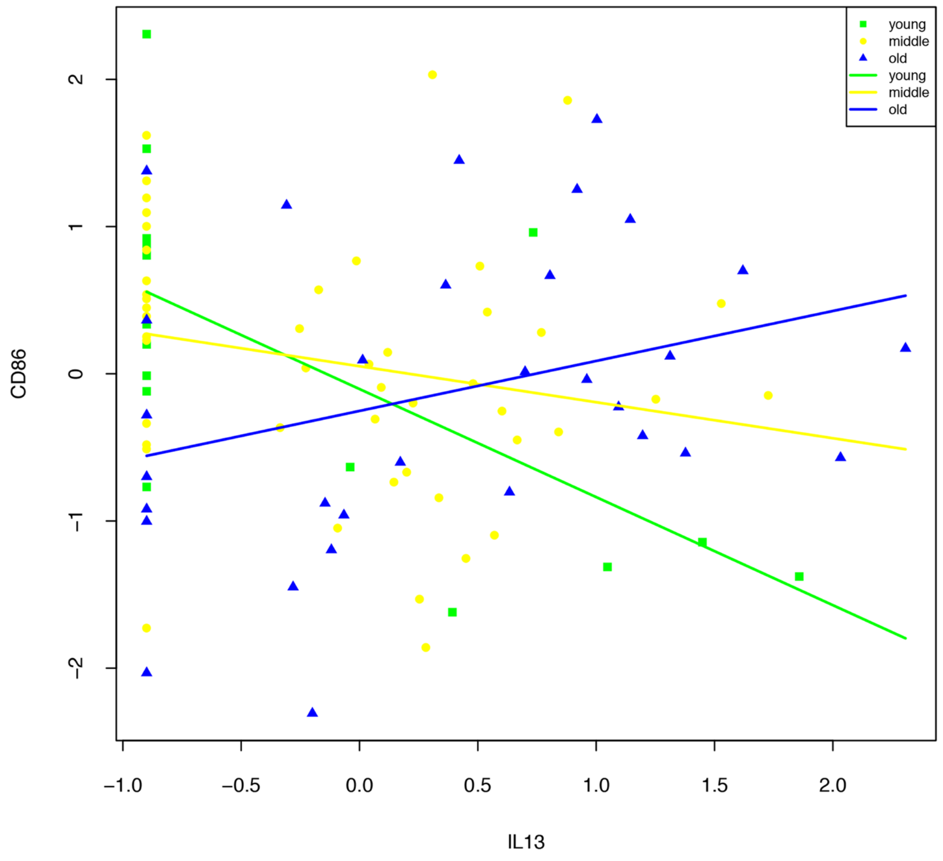

young
middle
old
young
middle
old
CD86
IL13

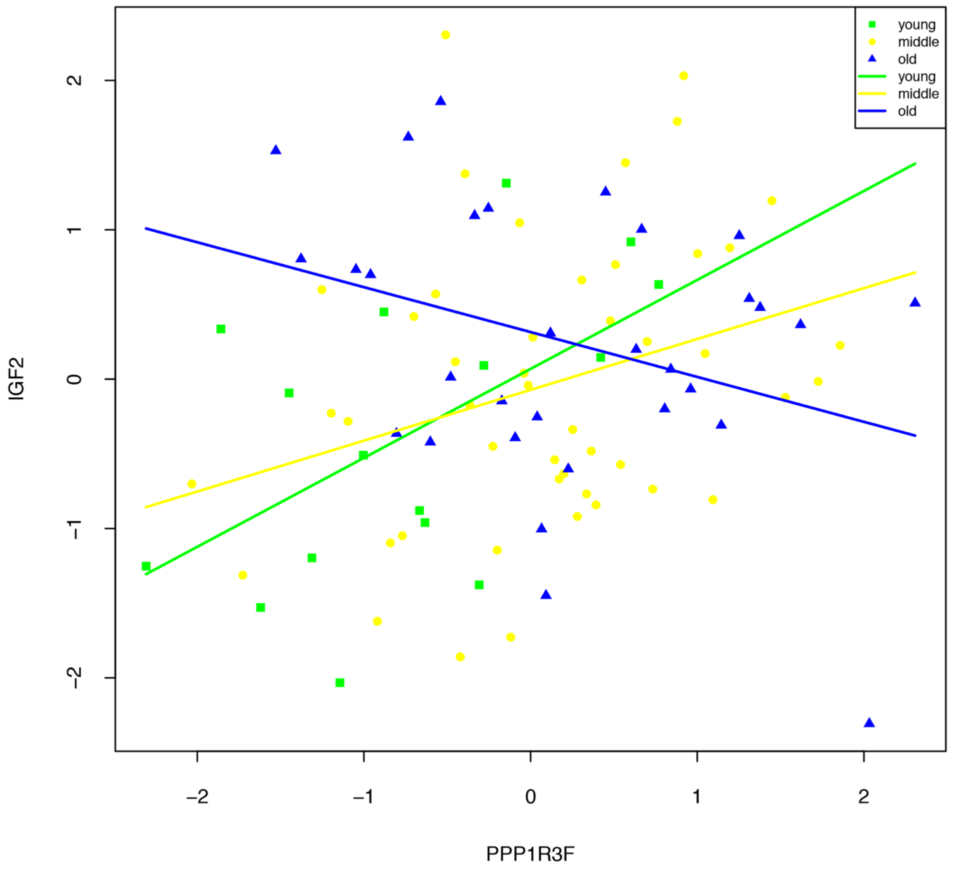

young
middle
old
young
middle
old
IGF2
PPP1R3F

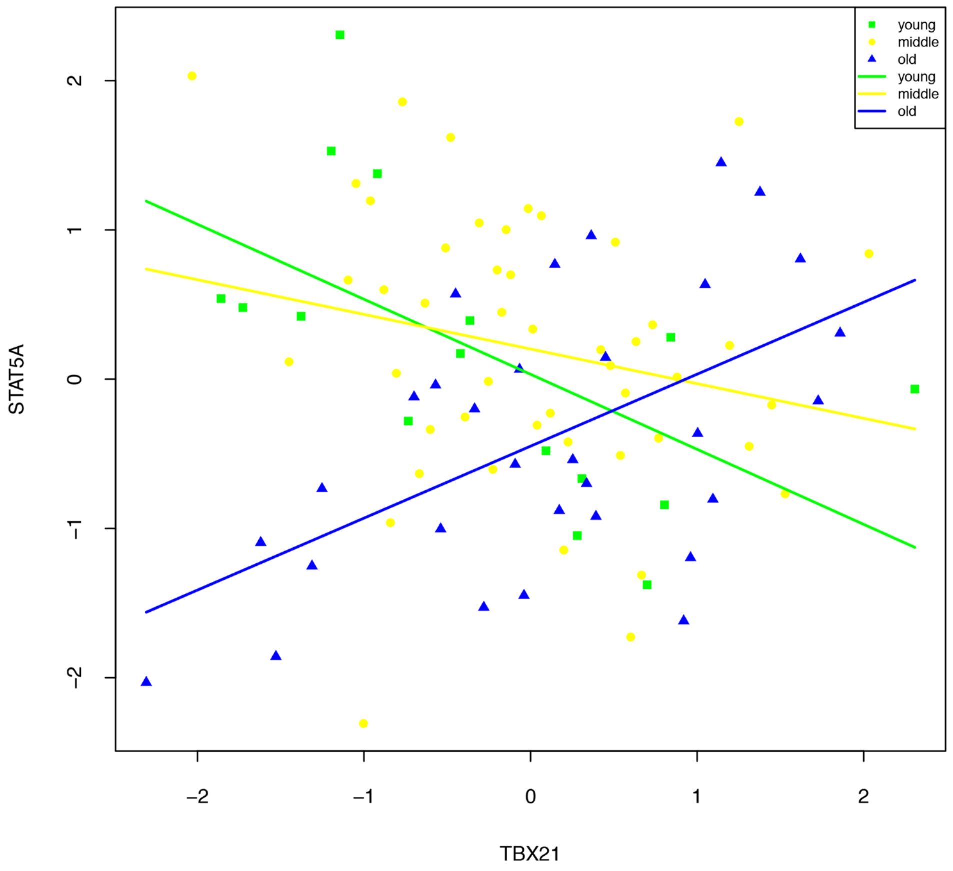
young
middle
old
young
middle
old
STAT5A
TBX21
−2
−1
0
1
2

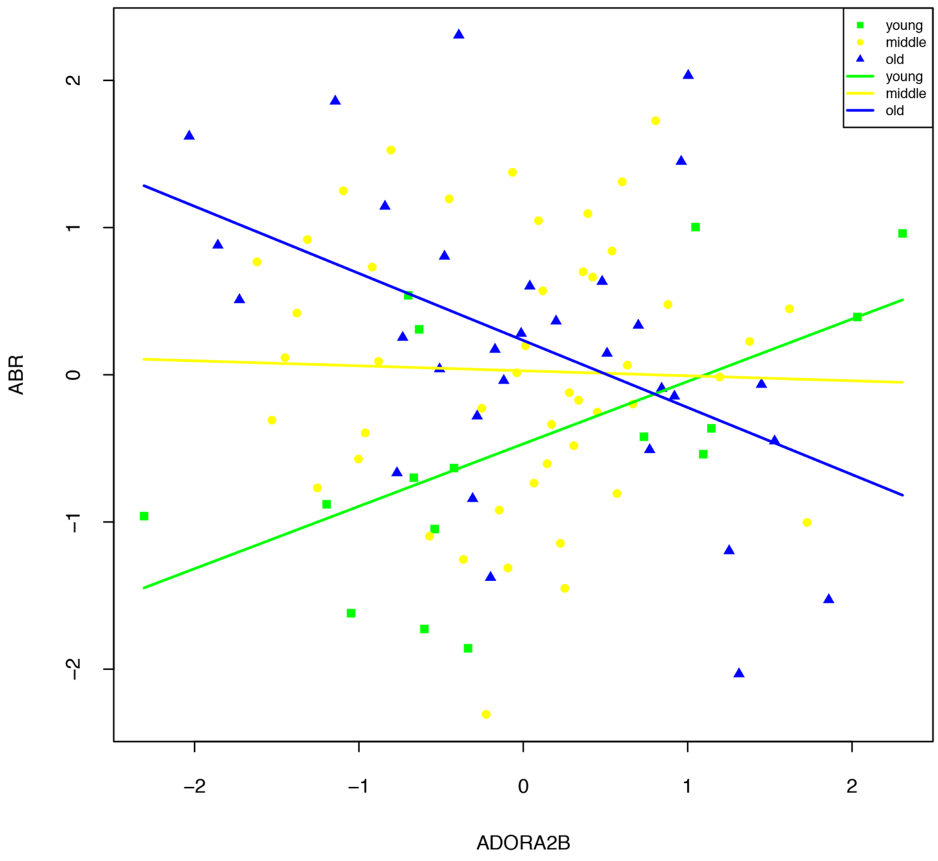
young
middle
old
young
middle
old
ABR
ADORA2B
−2
−1
0
1
2

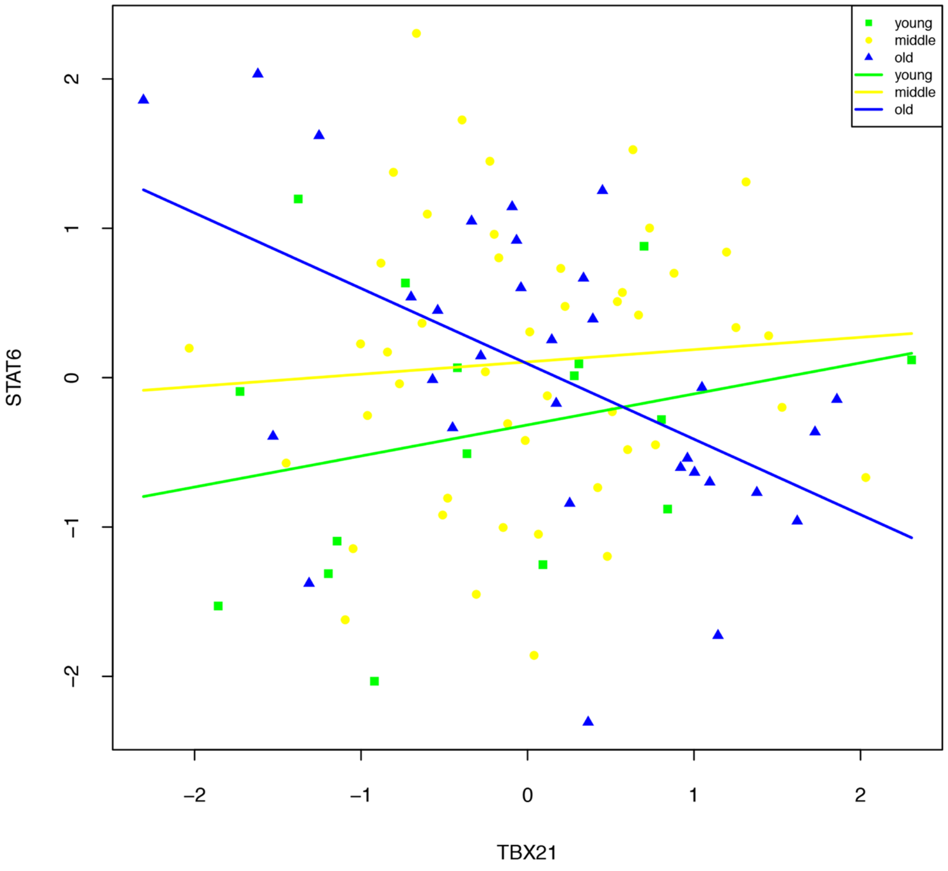
young
middle
old
young
middle
old
STAT6
TBX21
2
1
0
−1
−2
−2
−1
0
1
2

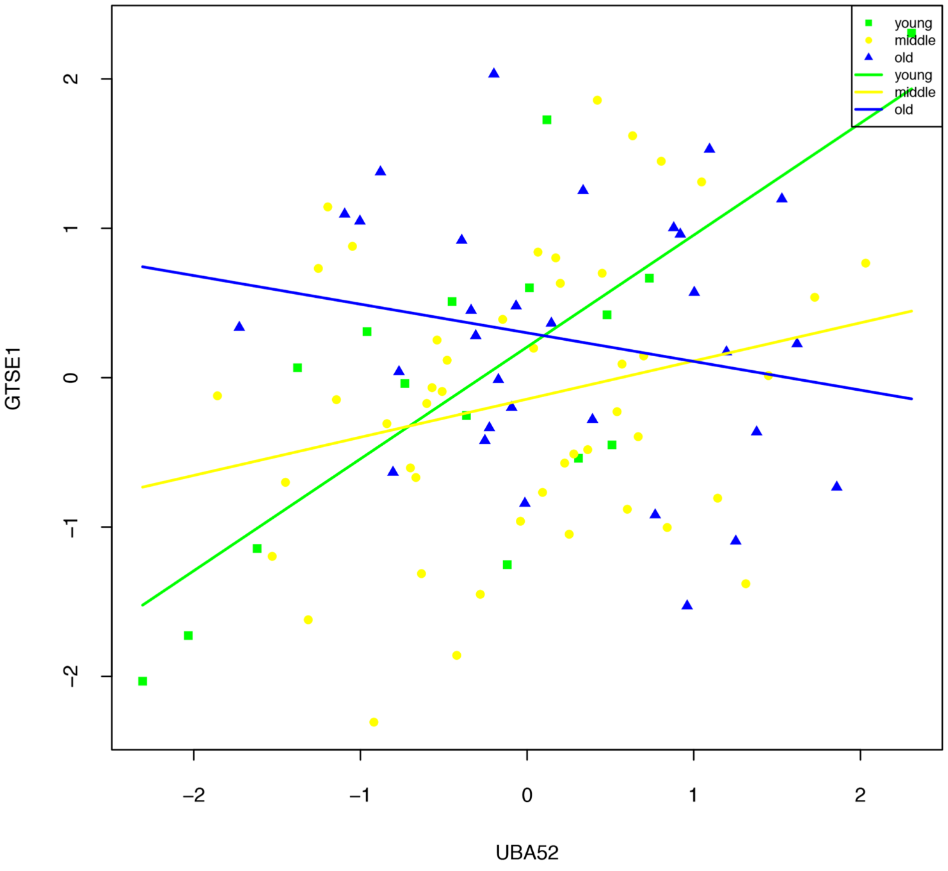
young
middle
old
young
middle
old
GTSE1
UBA52
2
1
0
−1
−2
−2
−1
0
1
2

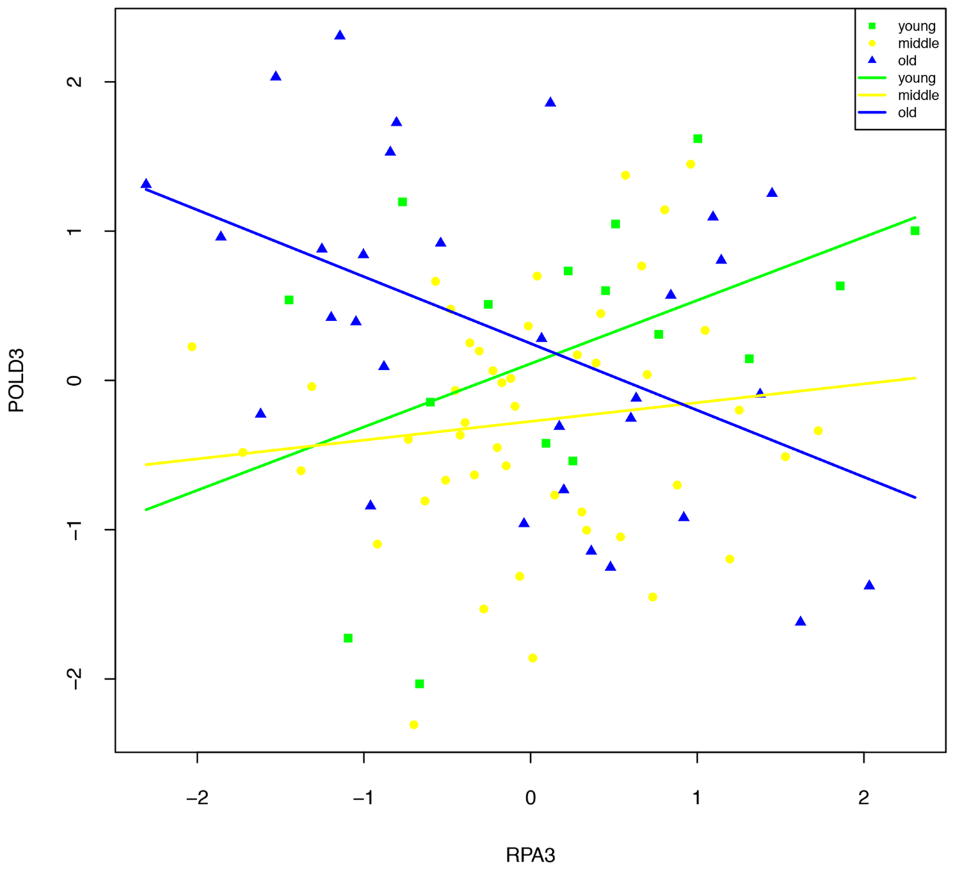
young
middle
old
young
middle
old
POLD3
RPA3
−2
−1
0
1
2

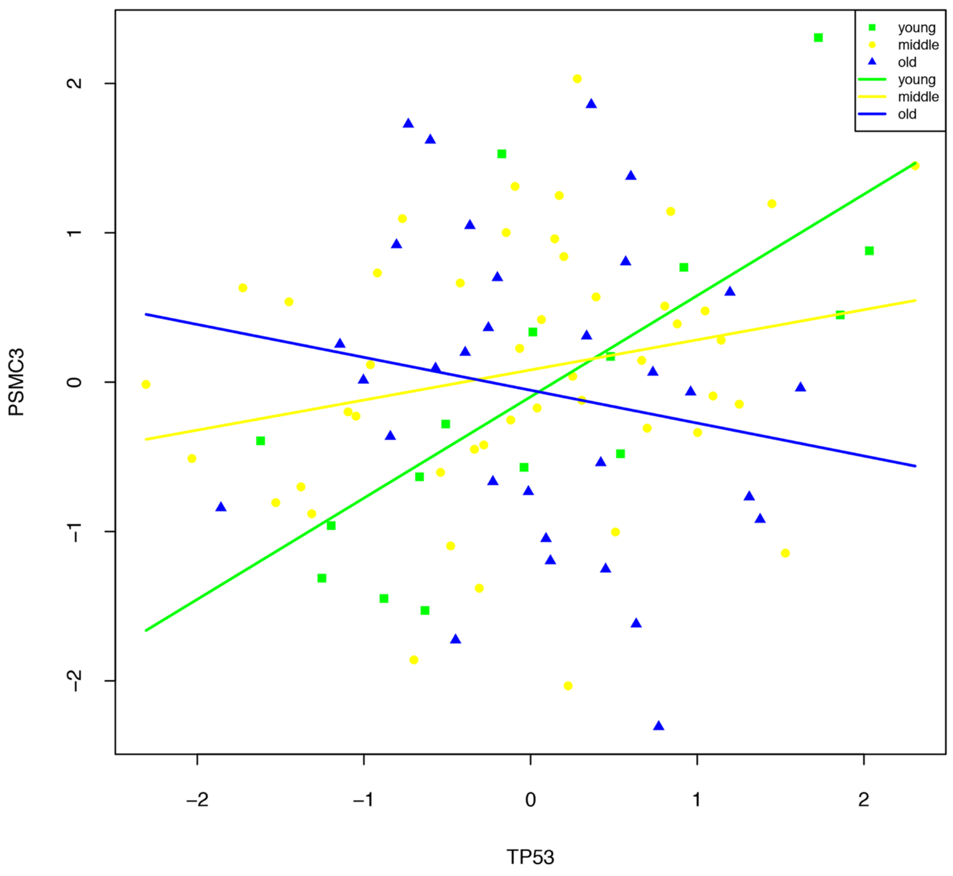
young
middle
old
young
middle
old
PSMC3
TP53
−2
−1
0
1
2

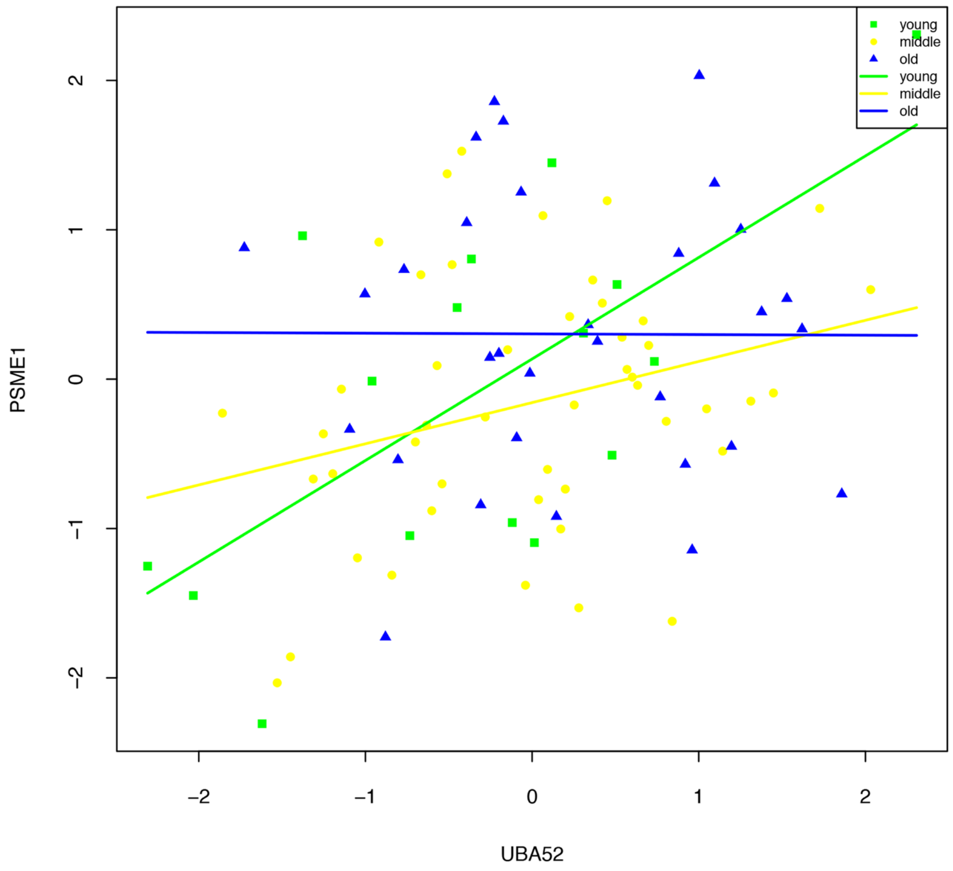
young
middle
old
young
middle
old
PSME1
UBA52
−2
−1
0
1
2

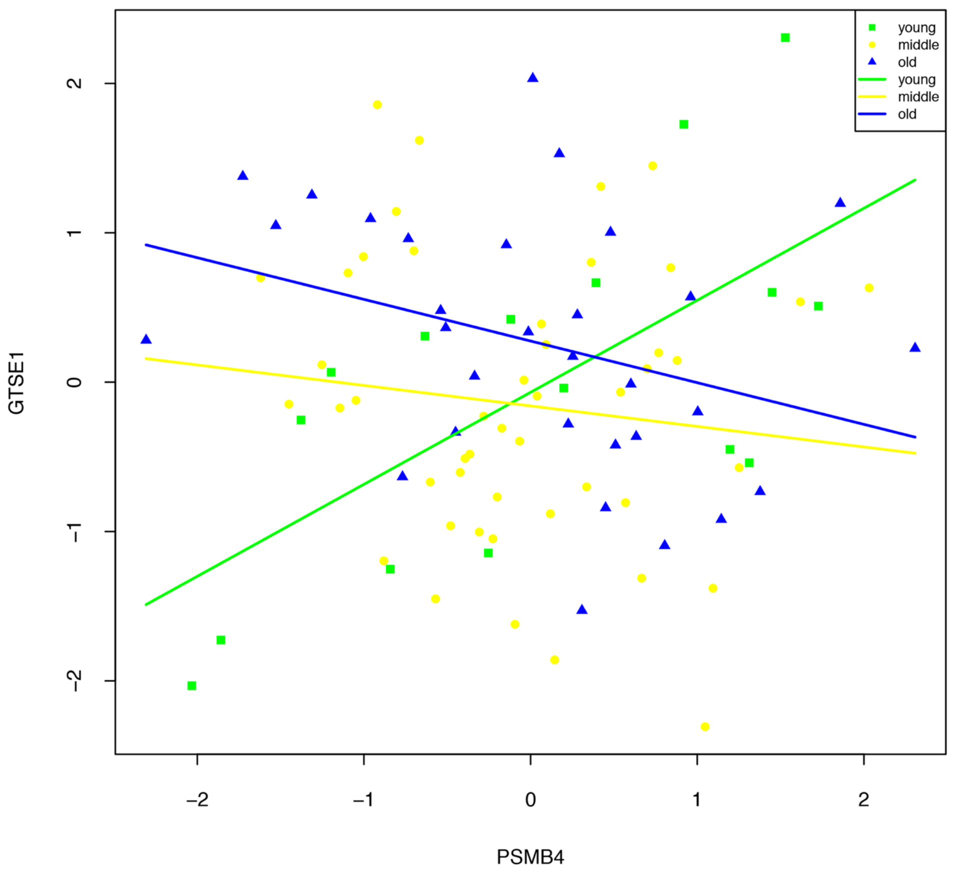
young
middle
old
young
middle
old
GTSE1
PSMB4
−2
−1
0
1
2

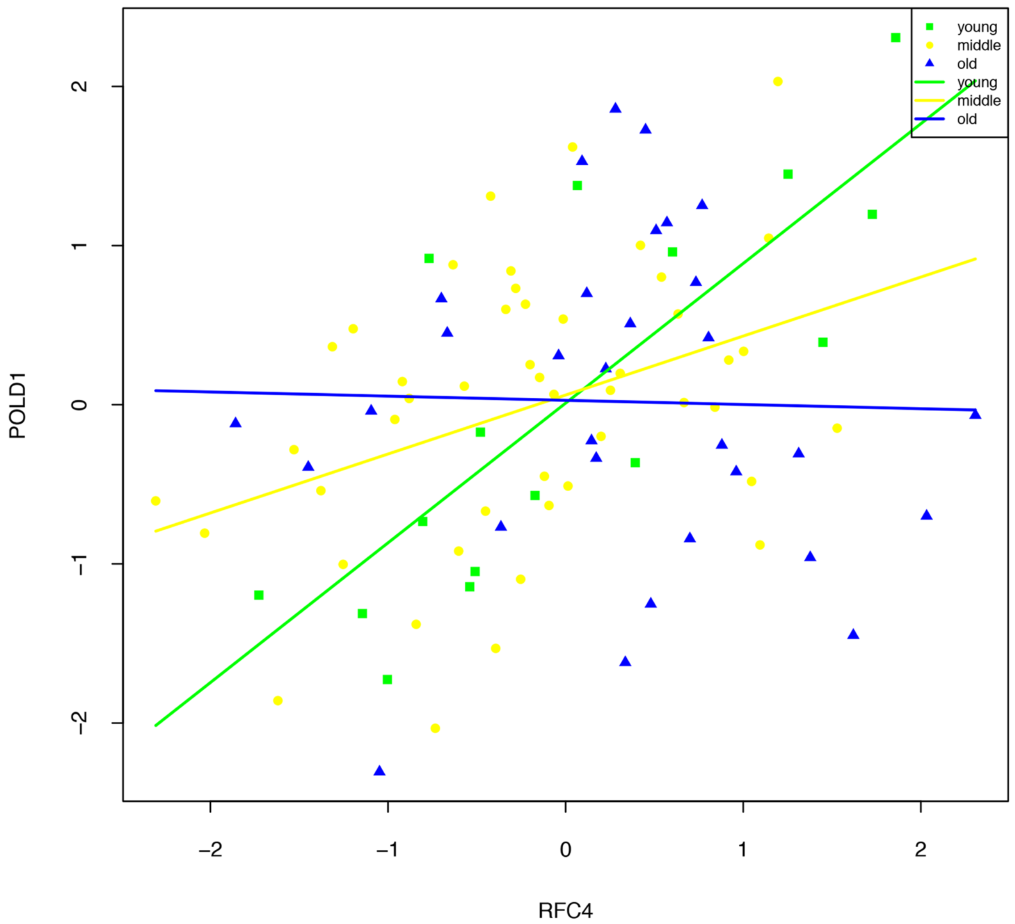

Supplementary Figure 1: Group-based gene-gene correlation of top 20 age-associated gene co-expressions for adipose tissue.

Supplementary Table 1: Liquid associated gene pairs sorted by their occurrences at GO and KEGG modules. See Supplementary_Table_1

Supplementary Table 2: Fisher exact test results of the detected gene pairs by liquid association and Pearson correlation coefficient. See Supplementary_Table_2

Supplementary Table 3: Functional annotations by the David tools for all the tissues. See Supplementary_Table_3

Supplementary Table 4: Results of testing the consistency of drug perturbation genes with significant up-regulated genes and down regulated genes for 4295 drugs. See Supplementary_Table_4
